# Father-to-offspring transmission of extremely long *NOTCH2NLC* repeat expansions with contractions: genetic and epigenetic profiling with long-read sequencing

**DOI:** 10.1186/s13148-021-01192-5

**Published:** 2021-11-13

**Authors:** Hiromi Fukuda, Daisuke Yamaguchi, Kristofor Nyquist, Yasushi Yabuki, Satoko Miyatake, Yuri Uchiyama, Kohei Hamanaka, Ken Saida, Eriko Koshimizu, Naomi Tsuchida, Atsushi Fujita, Satomi Mitsuhashi, Kazuyuki Ohbo, Yuki Satake, Jun Sone, Hiroshi Doi, Keisuke Morihara, Tomoko Okamoto, Yuji Takahashi, Aaron M. Wenger, Norifumi Shioda, Fumiaki Tanaka, Naomichi Matsumoto, Takeshi Mizuguchi

**Affiliations:** 1 Department of Human Genetics, Yokohama City University Graduate School of Medicine, 3-9 Fukuura, Kanazawa-ku, Yokohama, 236-0004 Japan; 2 Department of Neurology and Stroke Medicine, Yokohama City University Graduate School of Medicine, Yokohama, Japan; 3 BioInformation Technology & Science, Tokyo, Japan; 4 Pacific Biosciences, Menlo Park, CA USA; 5 Department of Genomic Neurology, Institute of Molecular Embryology and Genetics (IMEG), Kumamoto University, Kumamoto, Japan; 6 Graduate School of Pharmaceutical Sciences, Kumamoto University, Kumamoto, Japan; 7 Clinical Genetics Department, Yokohama City University Hospital, Yokohama, Japan; 8 Department of Rare Disease Genomics, Yokohama City University Hospital, Yokohama, Japan; 9 Department of Genomic Function and Diversity, Medical Research Institute Tokyo Medical and Dental University, Tokyo, Japan; 10 Department of Histology and Cell Biology, Yokohama City University Graduate School of Medicine, Yokohama, Japan; 11 Department of Neurology, Yokkaichi Municipal Hospital, Yokkaichi, Japan; 12 Department of Neuropathology, Institute for Medical Science of Aging, Aichi Medical University, Nagakute, Japan; 13 Department of Neurology, National Hospital Organization Suzuka National Hospital, Suzuka, Japan; 14 Department of Neurology, National Center Hospital, National Center of Neurology and Psychiatry, Tokyo, Japan

**Keywords:** Neuronal intranuclear inclusion disease, *NOTCH2NLC*, Repeat expansion, DNA methylation, Long-read sequencing, Single molecule epigenetic analysis

## Abstract

**Background:**

GGC repeat expansions in *NOTCH2NLC* are associated with neuronal intranuclear inclusion disease. Very recently, asymptomatic carriers with *NOTCH2NLC* repeat expansions were reported. In these asymptomatic individuals, the CpG island in *NOTCH2NLC* is hypermethylated, suggesting that two factors repeat length and DNA methylation status should be considered to evaluate pathogenicity. Long-read sequencing can be used to simultaneously profile genomic and epigenomic alterations. We analyzed four sporadic cases with *NOTCH2NLC* repeat expansion and their phenotypically normal parents. The native genomic DNA that retains base modification was sequenced on a per-trio basis using both PacBio and Oxford Nanopore long-read sequencing technologies. A custom workflow was developed to evaluate DNA modifications. With these two technologies combined, long-range DNA methylation information was integrated with complete repeat DNA sequences to investigate the genetic origins of expanded GGC repeats in these sporadic cases.

**Results:**

In all four families, asymptomatic fathers had longer expansions (median: 522, 390, 528 and 650 repeats) compared with their affected offspring (median: 93, 117, 162 and 140 repeats, respectively). These expansions are much longer than the disease-causing range previously reported (in general, 41–300 repeats). Repeat lengths were extremely variable in the father, suggesting somatic mosaicism. Instability is more frequent in alleles with uninterrupted pure GGCs. Single molecule epigenetic analysis revealed complex DNA methylation patterns and epigenetic heterogeneity. We identified an aberrant gain-of-methylation region (2.2 kb in size beyond the CpG island and GGC repeats) in asymptomatic fathers. This methylated region was unmethylated in the normal allele with bilateral transitional zones with both methylated and unmethylated CpG dinucleotides, which may be protected from methylation to ensure *NOTCH2NLC* expression.

**Conclusions:**

We clearly demonstrate that the four sporadic *NOTCH2NLC*-related cases are derived from the paternal GGC repeat contraction associated with demethylation. The entire genetic and epigenetic landscape of the *NOTCH2NLC* region was uncovered using the custom workflow of long-read sequence data, demonstrating the utility of this method for revealing epigenetic/mutational changes in repetitive elements, which are difficult to characterize by conventional short-read/bisulfite sequencing methods. Our approach should be useful for biomedical research, aiding the discovery of DNA methylation abnormalities through the entire genome.

**Supplementary Information:**

The online version contains supplementary material available at 10.1186/s13148-021-01192-5.

## Background

Neuronal intranuclear inclusion disease (NIID) is a progressive neurodegenerative disease characterized by various clinical manifestations, such as cognitive decline, peripheral neuropathy, autonomic dysfunction, encephalitic episodes, parkinsonism, and cerebellar ataxia (OMIM #603472). Histologically, the presence of eosinophilic hyaline intranuclear inclusions is the pathological hallmark of NIID. On neuroimaging, NIID is characterized by high-intensity signals in the corticomedullary junction on diffusion-weighted imaging (DWI) of magnetic resonance imaging (MRI). These are useful diagnostic markers for NIID [1]. In 2019, GGC repeat expansion in the 5ʹ-untranslated region (5ʹ-UTR) of *NOTCH2NLC* was identified as causative in familial and sporadic cases of NIID [2–4]. Now, genetic tests targeting *NOTCH2NLC* repeat-expansion, which have been widely used, have revealed a surprisingly broad clinical spectrum [5–10]. Furthermore, studies to understand the molecular basis of repeat instability and the pathomechanisms of GGC repeat expansion have just started [11–13]. In particular, three and two asymptomatic individuals with *NOTCH2NLC* repeat-expansion accompanied by DNA hypermethylation in this region were very recently reported in oculopharyngodistal myopathy (OPDM) and NIID, respectively [11, 12].

We recruited four affected individuals with *NOTCH2NLC*-related disorders whose trio-based mutation screening indicated possible de novo occurrence of GGC repeat expansion in previous reports. [3, 8–10]. The origin of new expansion mutations in these families remains unclear.

In typical repeat expansion diseases, the pathogenic repeats are more prone to expand during parent-to-offspring transmission, leading to increase in disease severity and/or earlier onset disease, in a phenomenon termed genetic anticipation. Such repeat instability is also a risk factor for new mutations in families with intermediate-size repeats (premutation), pointing to genetic changes being behind the molecular basis of clinical expression. Moreover, repeat instability is affected by cis- and trans-elements, such as repetitive sequences, repeat configurations, repeat compositions, interruption sequences, nearby sequence variations, CpG methylation, and replication origins [14]. As such, to better understand the possible de novo* NOTCH2NLC* mutations, accurate determination of whole and nearby repeat sequences is important.

In the present study, we investigate four sporadic cases with possible de novo mutations using long-read sequencing technologies to probe the genetic and epigenetic landscape of *NOTCH2NLC*.

## Results

### Long GGC repeat expansion in four asymptomatic fathers

**Fig. 1:**
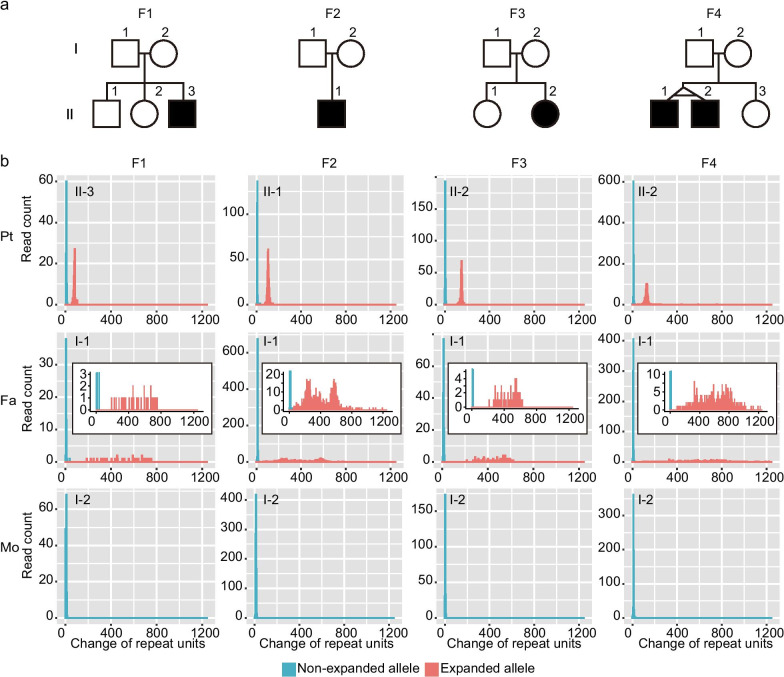
Repeat copy-number analysis of four sporadic cases and their parents. **a** Familial pedigree of four sporadic cases having *NOTCH2NLC* GGC expansion. **b** Repeat-size evaluation of *NOTCH2NLC* GGC repeats using tandem-genotypes. The copy number changes in the *NOTCH2NLC* GGC repeat relative to the human reference genome (hg38) were examined in the patient-parents trio in four families. Pale blue: non-expanded allele; pale pink: expanded allele. *Pt* patient; *Fa* father; *Mo* mother

Four affected individuals were previously reported to have possible de novo GGC repeat expansion of *NOTCH2NLC* (Fig. 1a). II-3 in Family F1 (ID3661 in a previous report) was diagnosed as sporadic NIID [3]. II-1 in Family F2 (Patient 11 in a previous report) had leukoencephalopathy [10]. II-2 in Family F3 was diagnosed as NIID with mitochondrial encephalomyopathy, lactic acidosis, and stroke-like episode (MELAS) in her clinical course of polyneuropathy [9]. II-2 in Family F4 is an affected twin with oculopharyngodistal myopathy (OPDM) (patient 7 in a previous report) [8]. The clinical features of these subjects are summarized in Additional file 2: Table S1, and have been previously described in detail [3, 8–10]. All fathers and mothers of the four affected individuals were enrolled and examined genetically in a per-trio basis, and are an invaluable resource in such late-onset adult diseases.

To investigate the genetic basis underlying these possible de novo mutations, trio samples (patient plus parents) of all four families were sequenced by targeting the 4-kb genomic region (chr1:149389497-149393469 in hg38) by Cas9-mediated PCR-free enrichment with Nanopore long-read sequencing [3]. We analyzed Cas9-mediated enrichment sequencing data by tandem-genotypes to evaluate the number of GGC repeat units in *NOTCH2NLC* [15]. Tandem-genotypes was able to discriminate non-expanded and expanded alleles with different repeat copy-numbers. As expected, all four patients—II-3 (F1-patient), II-1 (F2-patient), II-2 (F3-patient) and II-2 (F4-patient)—had disease-causing GGC expansions with median repeat copy-numbers of 93, 117, 162 and 140, respectively [80, 104, 149 and 127, respectively, relative to the reference (13 copies) in tandem-genotypes output], whereas there were no expanded alleles in their healthy mothers (Fig. 1b and Additional file 2: Table S2). Despite negative results of repeat-primed polymerase chain reaction (RP-PCR), we noticed expanded alleles in all paternal sequencing samples (Fig. 1b and Additional file 1: Fig. S1). Unexpectedly, the repeats in the paternal samples were much longer than those in their affected offspring and were extremely variable in repeat length (Fig. 1b and Additional file 2: Table S2). Such high variability had never been seen before, but the substantial numbers of reads (39, 1070, 88 and 505 reads for F1-father, F2-father, F3-father and F4-father, respectively) supported the presence of expansion alleles (Additional file 2: Table S2). To confirm this result, we performed Southern blot analysis using a probe near the *NOTCH2NLC* GGC repeat, and confirmed the long repeat expansion in paternal samples in three families (Southern blot analysis could not be performed in F1 family because of insufficient amount of DNA) (Additional file 1: Fig. S2). The four fathers were clinically asymptomatic based on careful interviews by professional neurologists, suggesting that the fathers are asymptomatic carrier males with extremely long *NOTCH2NLC* repeat expansion.

### Complete GGC repeat expansion sequence

**Fig. 2:**
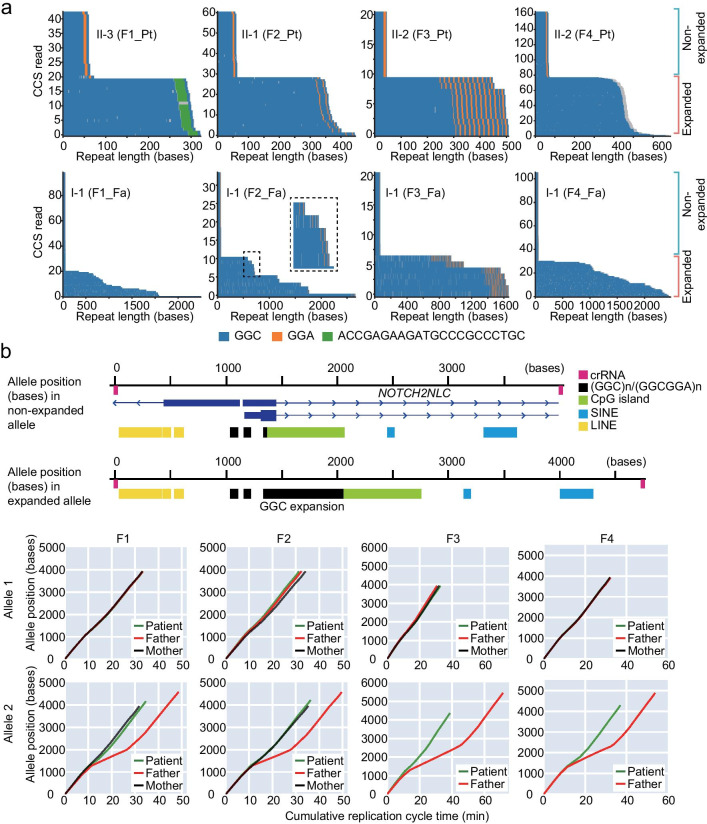
Repeat sequence content and polymerase kinetics using PacBio HiFi sequencing. **a** Waterfall plots showing complete repeat structure of non-expanded, pathogenic and non-pathogenic expanded alleles excised by the CRISPR/Cas9-based enrichment method (No-Amp) in patients and their asymptomatic fathers. Y-axis shows the number of circular consensus sequence (CCS) reads, whereas the X-axis shows the length of the repeat expansions in bases. GGC, GGA and ACCGAGAAGATGCCCGCCCTGC sequences are shown as blue, orange and green short longitudinal lines, respectively. **b** Upper line shows cas9-targeted region with RefSeq and repeatmasker annotation from UCSC genome browser (https://genome.ucsc.edu/). Lower graphs show polymerase kinetics of non-expanded and expanded alleles for each allele during the SMRT sequencing. x-axis: cumulative replication cycle time; y-axis: numbers represent the base pair position within cas9-excised DNA fragment for each allele (allele position). Allele 1: non-expanded allele; Allele 2: expanded allele of patients and their fathers or second non-expanded allele of the F1-mother and F2-mother. Unphased non-expanded alleles of the F3-mother and F4-mother are displayed in allele 1 because the two non-expanded alleles had similar repeat sizes and could not be separated. *Pt* patient; *Fa* father; *Mo* mother. Magenta, black, green, blue and yellow rectangles represent crRNAs, (GGC)n/(GGCGGA)n, CpG island, SINE and LINE repetitive elements, respectively

We next completely sequenced the GGC repeat expansion. Given the high deletion and insertion error rates in Nanopore sequencing, constructing the consensus sequence from multiple Nanopore reads (i.e., inter-molecular consensus) is preferable. However, repeat length was too variable to generate an inter-molecular consensus sequence (Fig. 1b). To overcome this difficulty, we used the PacBio no-amplification (No-Amp) targeted sequencing method, which can generate high-fidelity consensus reads (HiFi reads) from multiple passes of subreads taken from a single template molecule (i.e., intra-molecular consensus) [16]. Indeed, the No-Amp method uncovered the whole expansion sequence in the paternal samples, which had not been well characterized before (Fig. 2a and Additional file 2: Table S3). The repeat size in each HiFi read varied, again indicating repeat instability and/or possible somatic mosaicism of long expanded alleles, as suggested by Nanopore analysis (Fig. 1b and Additional file 2: Table S2). The F1-father and F4-father had pure (GGC)n configuration, whereas the F2-father and F3-father had the (GGC)n followed by [(GGA)_n_(GGC)_n_]_n_ repeats (Fig. 2a and Additional file 2: Table S3). Importantly, patients had the same repeat configurations as their fathers, although repeat size was contracted, indicating the paternal origin of the pathogenic allele (Fig. 2a). Consistently, all non-expanded alleles were transmitted from their mothers, as indicated by amplicon-length PCR (AL-PCR) analysis (Additional file 1: Fig. S3).

### Detection of DNA modification by PacBio SMRT sequencing

**Fig. 3:**
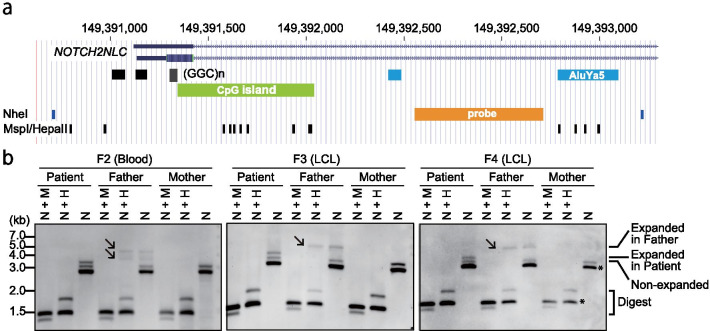
Southern blot analysis showing the differential DNA methylation status of the *NOTCH2NLC* region. **a** Physical map of the *NOTCH2NLC* region with the NheI, MspI/HpaII restriction enzyme sites and Southern blot probe. Chromosome positions are based on hg38 assembly. Black, green, blue and orange rectangles represent (GGC)n/(GGCGGA)n, CpG island, SINE and Southern blot probe, respectively. **b** Genomic DNA extracted from blood samples (F2 family) and LCLs (F3 and F4 families) were digested by the restriction enzyme indicated in the figure panel. N + M: NheI and MspI; N + H: NheI and HpaII; N: NheI. Note that expanded alleles in the father’s sample are resistant to restriction digestion by the methylation-sensitive endonuclease HpaII (arrow). Asterisk: cross hybridization to the *NOTCH2NLC* homologous genes, *NOTCH2*, *NOTCH2NLA*, *NOTCH2NLB* and *NOTCH2NLR*. Note that two prominent signals (arrows) were detected in the blood sample from the F2-father, but only one signal (arrow) was detected in LCLs of the F3-father and F4-father, indicating somatic mosaicism

As described above, the disease-causing allele was inherited from their asymptomatic carrier fathers. The lack of clinical symptoms in the carrier fathers indicates differences in the functional consequences of GGC expansion between patients and carrier fathers. We previously reported that there is no DNA methylation difference between expanded and non-expanded alleles in NIID patients [3]. Therefore, we were curious about the methylation status of the asymptomatic carrier fathers. We examined the base modification, which can be inferred from measuring the kinetics of replication during the PacBio single-molecule real-time (SMRT) sequencing run (polymerase kinetics), using the No-Amp data. Intriguingly, base incorporation in the GGC repeat region (i.e., DNA polymerase speed) was much slower in the four asymptomatic carrier fathers (0.9 bases/second in the repeat expansion allele vs. 2.0 bases/second in the non-expanded allele), suggesting DNA hypermethylation in the repeat expansion allele (Fig. 2b and Additional file 1: Fig. S4). To confirm this observation, we performed Southern blot analysis using the methylation-sensitive restriction enzyme HpaII and the methylation-insensitive restriction enzyme MspI. Genomic DNA was initially digested with NheI, and then the DNA methylation status was compared by examining the HpaII and MspI digestion efficiencies of the GGC repeat-containing NheI fragment (Fig. 3a). The NheI DNA fragments with the GGC repeats were completely resistant to HpaII digestion in the fathers’ samples, but not in patients or mothers, confirming the presence of fully hypermethylated CpG in all three fathers tested (Southern blot analysis could not be performed in family F1 because of insufficient DNA) (Fig. 3b).

### DNA methylation landscape of the *NOTCH2NLC* region assessed by Nanopore sequencing

To investigate the hypermethylated region at a higher resolution, we developed a custom program to detect 5-mC from Nanopore sequencing. Our analysis was based on Guppy, a basecalling program from Oxford Nanopore Technologies, which directly identifies 5-mC by calculating the likelihood of base modification [17]. The modified base information from Guppy was assigned to the genomic position by our custom program methylstat, and used for methylation calling at that genomic position using methylcall. This modified base information was also used for generating BAM files containing the in silico bisulfite-like base-converted reads for IGV visualization (ont2bisul) (see the Methods section for further detail).

Our analysis revealed heterozygous gain of 5-mC in the father of patient F2 (F2-father) which can be properly detected by our methylation calling method methylcall (Additional file 1: Fig. S5). A comparison of the methylation calls between the patient (F2-patient) and his father (F2-father) revealed that the 2.2 kb genomic region was hypermethylated en bloc, encompassing regions 700-bp upstream and 1,000-bp downstream of the GGC expanded repeat (Additional file 1: Fig. S5).

**Fig. 4:**
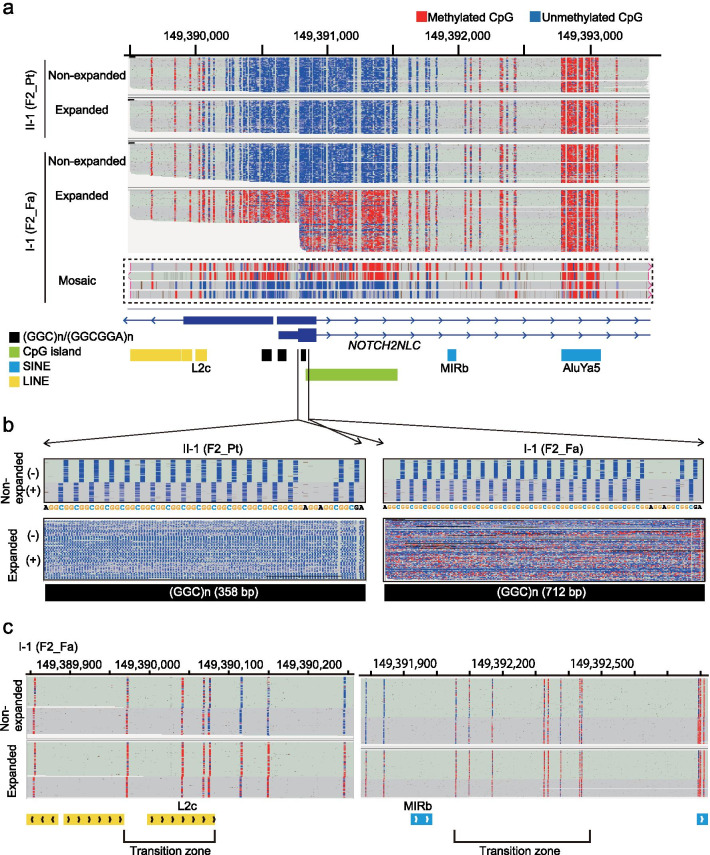
Detailed DNA methylation landscape of the *NOTCH2NLC* region by nanopore sequencing. **a** Haplotype-resolved methylation analysis of the 4-kb *NOTCH2NLC* region in the F2 family. Blue: unmethylated CpG; red: methylated CpG. Representative reads with hypo- and hyper-methylated expansion alleles, indicating epigenetic mosaicism, are shown in the lower inset (dashed-square). **b** DNA methylation status within the *NOTCH2NLC* repeat. Reads were sorted by read strand. + : forward reads ( +); − : reverse reads ( −). **c** DNA methylation status of transitional zones. Long-range methylation information of individual Nanopore reads was visualized with the IGV bisulfite mode. Chromosome positions are according to the hg38 assembly. *Pt* patient; *Fa* father; *Mo* mother. Black, green, blue and yellow rectangles represent (GGC)n/(GGCGGA)n, CpG island, SINE and LINE, respectively, from UCSC genome browser

Taking advantage of long-read sequencing, we performed haplotype-phasing based on GGC repeat copy-number from the tandem-genotypes results. This analysis clearly showed that only the non-pathogenic long expansion allele in asymptomatic fathers, but not the disease-causing expansion allele in patients, was hypermethylated (Fig. 4a). The read-level plot also enabled us to observe the methylation status of individual DNA molecules, confirming that some reads with long expansion were not hypermethylated, indicating epigenetic mosaicism (Fig. 4a, inset).

### DNA methylation status within the GGC repeat sequence

We next investigated the 5-mC status of the *NOTCH2NLC* GGC repeat sequence using a reference-free approach. This requires the entire repeat expansion DNA sequence and the corresponding methylation information for the subject. Taking advantage of two technologies, PacBio and Nanopore, we generated high-quality consensus GGC repeat sequences from PacBio HiFi reads (Additional file 1: Fig. S6), and then mapped the 5-mC annotated nanopore reads to their respective GGC repeat expansion sequence (HiFi consensus as the expansion reference). Not all, but a proportion of CpG sites in the long GGC expansion in the asymptomatic father (F2-father) had the 5-mC modification, whereas the disease-causing expansion allele in the patient (F2-patient) was completely unmethylated (Fig. 4b).

### DNA methylation boundary

A region spanning approximately 700 bases upstream and 1,000 bases downstream of the GGC repeat was completely unmethylated (chr1: 149390115-149391841) in the non-expanded allele (Fig. 4a and Additional file 1: Fig. S7). The region outside of this unmethylated region was hypermethylated (Fig. 4a and Additional file 1: Fig. S7). We identified two distinct transitional zones between the completely unmethylated and hypermethylated regions. The methylation status of these transitional zones consisted of methylated and unmethylated CpGs, indicative of DNA methylation mosaicism, similar to the DNA methylation boundary in the FMRP translational regulator 1 (*FMR1*) promoter [18] (Fig. 4c). Four (chr1: 149389972-149389973, 149390041-149390041, 149390068-149390069 and 149390075-149390076) and eight (chr1: 149392058-149392059, 149392099-149392100, 149392169-149392170, 149392326-149392327, 149392338-149392339, 149392376-149392377, 149392434-149392435 and 149392440-149392441) CpG dinucleotides were characterized by a mosaic pattern of DNA methylation, upstream and downstream of the GGC repeat (Fig. 4c). The position of the transition zones was conserved in all 12 individuals tested in this study (Additional file 1: Fig. S7). These transitional zones were lost in the repeat-expanded allele in the four asymptomatic fathers, and the CpG dinucleotides were fully methylated (Fig. 4c).

In summary, the combination of the two long-read sequencing technologies allowed us to characterize the genomic and epigenomic landscape of the pathogenic (disease-causing) and non-pathogenic *NOTCH2NLC* repeat regions.

### Pathological cell-context consequences of pathogenic *NOTCH2NLC* repeat expansion

**Fig. 5:**
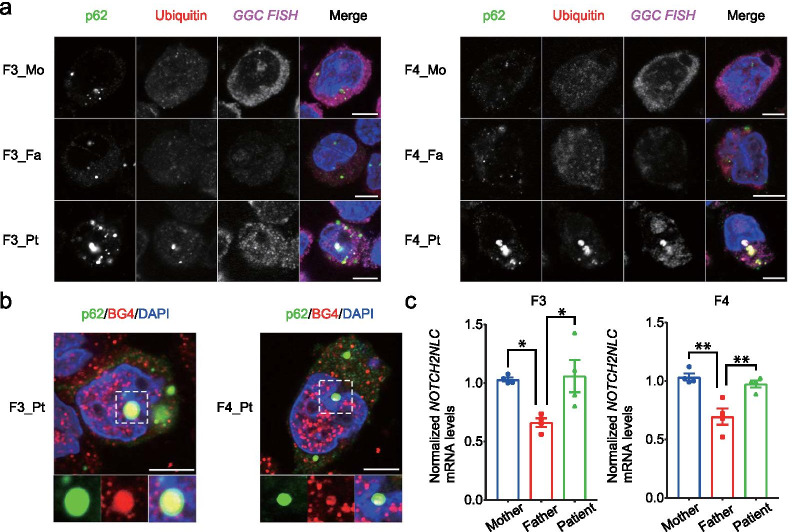
Pathological study of *NOTCH2NLC* GGC repeat expansion. **a** immunofluorescence and RNA FISH experiment showing nuclear inclusions with GGC RNA foci. Representative images of p62 (green), ubiquitin (red), fluorescent probe of (GGC)_8_ (magenta) and DAPI (blue) in LCLs derived from F3 (left) and F4 (right) families. **b** Immunofluorescence experiment showing co-localization of G4 foci and intranuclear inclusions. Representative images of p62 (green), BG4 (red) and DAPI (blue) in LCLs of the F3-patient and the F4-patient. All scale bars: 5 μm. **c** RT-qPCR experiment for *NOTCH2NLC* expression. mRNA levels of *NOTCH2NLC* were significantly decreased in LCLs from each father compared with the corresponding mother and the corresponding patient (*n* = 4 per group). Error bars represent SEM. *p < 0.05, **p < 0.01

As described above, we observed differential DNA methylation between patients and their asymptomatic fathers. Gain of DNA methylation in asymptomatic carriers likely suppresses the toxic effect of the long GGC expansion allele. To dissect the pathological cell-context consequences of disease-causing and non-pathogenic long expansions, we investigated the formation of nuclear inclusion bodies using immunocytochemistry combined with FISH for detecting GGC-repeat expansion RNA in lymphoblastoid cell lines (LCLs) derived from F3 and F4 families. Approximately 3% of LCLs showed ubiquitin- and p62-double positive intranuclear inclusions in both the F3-patient and F4-patient, with the disease-causing allele, but not in their healthy mothers or asymptomatic fathers (Fig. 5a, Additional file 2: Table S4). Importantly, intranuclear inclusions were negative in fathers with extremely long expanded GGCs, indicating the distinct pathological consequences of two different classes of GGC expansion among fathers and their affected offspring. These intranuclear inclusions were co-localized with FISH-labeled CGG-RNA (only in affected offspring), suggesting that *NOTCH2NLC* mRNA with disease-causing GGC expansions was not only distributed diffusely in the nucleus, but also co-aggregated with the inclusions, as reported previously on skin biopsy samples [12]. These observations confirmed that fathers are indeed clinicopathologically normal.

Fragile X-associated tremor/ataxia syndrome (FXTAS), which is caused by a CGG repeat expansion in the 5ʹ-UTR region of *FMR1*, and NIID display striking similarities in clinical features and histological findings of intranuclear inclusions [19]. The CGG repeat expansion in *FMR1* mRNA results in the formation of G4 structures, which co-aggregate in intranuclear inclusions in brain tissues in FXTAS model mice [20]. Notably, we found that immunoreactivities of G4 foci co-localized with p62-positive intranuclear inclusions, suggesting the involvement of RNA G4 in NIID development in humans (Fig. 5b).

Next, we investigated the expression of *NOTCH2NLC* mRNA by qPCR in LCLs derived from F3 and F4 families. Consistent with DNA methylation analysis, expression of *NOTCH2NLC* mRNA in LCLs from both asymptomatic fathers was significantly decreased compared with the corresponding healthy mothers and patients (F3 father: 0.66 ± 0.038 (relative to F3 mother) vs. F3 mother (*p* < 0.05) and F3 patient (p < 0.05); F4 father: 0.70 ± 0.070 (relative to F4 mother) vs. F4 mother (*p* < 0.01) and F4 patient (p < 0.01) in Fig. 5c). Transcriptional repression of *NOTCH2NLC* in fathers was also supported by the relatively lower nuclear signal intensity of GGC RNA FISH (Fig. 5a). These evidences indicate extremely long expanded GGCs in fathers promote epigenetic changes that silence *NOTCH2NLC*. Only two families were studied in this study. Hence, further investigation with new families is needed to confirm the relationship between DNA methylation and *NOTCH2NLC* mRNA expression.

### Simple and fast identification of biologically important differentially methylated regions

Our methylation calling method (methylcall) can correctly detect hypermethylated bases, and is potentially useful for medical research for identifying differentially methylated regions among samples. However, methylcall cannot quantitatively measure the methylation status (Additional file 1: Fig. S5). Given the zygosity and epigenetic mosaicism, we decided to establish a quantitative method for Nanopore methylation data analysis. We calculated the methylation level by counting the ratio of 5-mC/(5-mC + C) at each base using a custom script (mtcall2mtkit), and analyzed it using Methylkit, which was originally developed for short-read methylation sequencing [21].

**Fig. 6:**
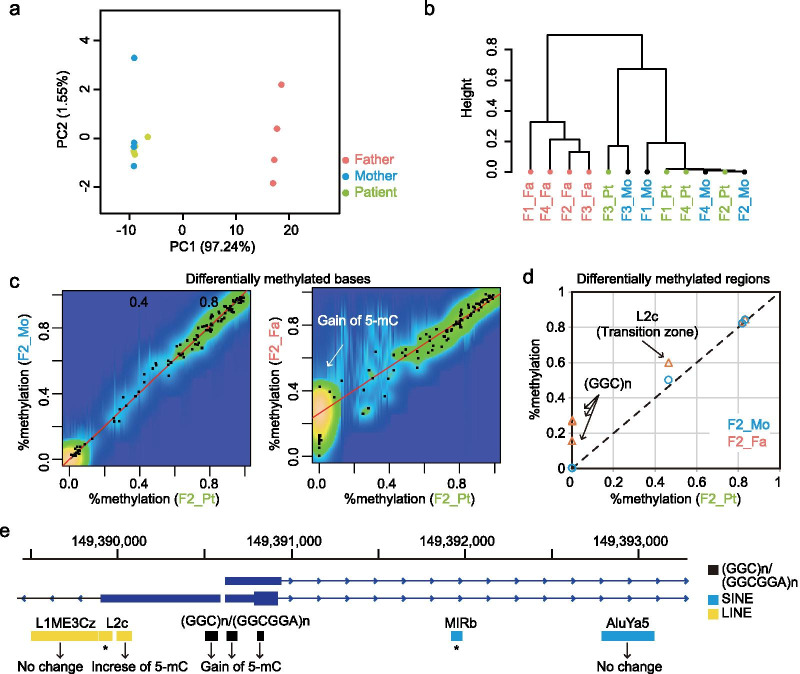
Multi-sample comparison of long-read methylation profiles. **a** Principal component analysis (PCA) of the 12 family members of sporadic cases with *NOTCH2NLC*-related disorders. **b** Hierarchical clustering analysis (Ward method) of the 12 family members of sporadic cases with *NOTCH2NLC*-related disorders. **c** Differentially methylated bases among samples. Pairwise comparison of percent methylation scores between samples are shown. Each dot represents the percent methylation scores of CpG sites at that genomic base position. The simple linear regression line plot in red. Pearson’s *r* = 0.998, with *p*-value < 2.2e − 16 (left panel); Pearson’s *r* = 0.955, with *p*-value < 2.2e − 16 (right panel). **d** Differentially methylated regions among samples. Differentially methylated bases are summarized in RepeatMasker annotation context. Percent methylation score of F2-patient was compared to F2-mother (blue open circle) or F2-father (red open triangle). **e** Schematic representation of differentially methylated region at the *NOTCH2NLC* region. Long-range methylation analysis can evaluate the methylation status of repetitive elements localized specifically at this region. Asterisk: not studied because of no CpG dinucleotides. Black, blue and yellow rectangles represent (GGC)n/(GGCGGA)n, SINE, and LINE repetitive elements, respectively. *Pt* patient; *Fa* father; *Mo* mother

The quantitative measurement enabled us to compare methylation levels across samples. As described above, single-sample analysis revealed the detailed methylation profile of the *NOTCH2NLC* region in the F2 family (Fig. 4). The other three families (F1, F3 and F4) had similar DNA methylation signatures (Additional file 1: Fig. S7). This similarity was confirmed by principal component analysis and hierarchical clustering analysis, revealing two clusters, one from asymptomatic carrier fathers and the other from patients and their mothers (Fig. 6a, b).

Methylkit can also extract and visualize differentially methylated bases between samples. As expected, the percent methylation values between the F2-mother and F2-patient had high similarity (Pearson’s correlation score of 0.998) (Fig. 6c). By contrast, the F2-father and F2-patient showed relatively less similarity (Pearson’s correlation score of 0.955) because a proportion of the differentially methylated cytosines were unmethylated in the F2-patient and methylated in the F2-father with the percent methylation values of mostly < 0.5, indicating heterozygous gain of 5-mC in the father (Fig. 6c and Additional file 2: Table S5).

Such differential DNA methylation is more useful when it is analyzed in the genomic context, such as repeats, CpG islands and promoters. We summarized percent methylation scores based on the RepeatMasker annotation from UCSC (http://genome.ucsc.edu/). In general, these regions are difficult to analyze by conventional methods using short-read next-generation sequencing because its repetitive nature hampers correct mapping of reads. Long-read sequencing has the advantage of being able to distinguish these repetitive elements from other copies throughout the human genome by spanning entire repeat sequences and adjacent unique regions as well as together with 5-mC. Moreover, these differentially methylated regions were evaluated in the context of gene annotation, such as the distance to transcription start site (TSS) and nearest gene name using Methylkit (Additional file 2: Table S6) [21]. Indeed, our analysis enabled the simple and fast identification of differentially methylated GGC repeats at *NOTCH2NLC* (Fig. 6d, e and Additional file 2: Table S6).

## Discussion

Two previous studies demonstrated that very long expansion in five asymptomatic carrier males, of which four were the fathers of affected individuals, was likely transmitted to sporadic cases showing *NOTCH2NLC*-related disorders [11, 12]. The CpG islands of *NOTCH2NLC* in the long expansion of carrier males have been shown to be hypermethylated, along with silencing of *NOTCH2NLC* transcription [11, 12]. Our current study not only provides an additional four cases to corroborate these observations, but also provides a more complete understanding of the genetic and epigenetic landscape of *NOTCH2NLC* at the nucleotide resolution using the power of single molecule epigenetics.

Here, four apparently sporadic affected individuals with *NOTCH2NLC* repeat expansion were investigated. Unexpectedly, the two long-read sequencing technologies (PacBio and Nanopore) uncovered the extremely long repeat-expansion alleles in paternal samples in all four families, despite negative results of previous RP-PCR based studies. Therefore, we repeated the RP-PCR experiment. Again, the longer expansion alleles in fathers were not amplified, whereas the pathogenic repeat-expansion alleles in the affected individuals were readily detected by RP-PCR (Additional file 1: Fig. S1). We note the abnormally slow polymerase kinetics (presumably due to 5-mC) in the long repeat expansion alleles by SMRT sequencing (Fig. 2b), which may be related to the inefficient RP-PCR amplification in fathers. Thus, PCR-free (single molecule) long-read sequencing technologies may be advantageous for evaluating *NOTCH2NLC* repeat expansion. Nonetheless, our findings provide additional evidence that the disease-causing allele in sporadic cases is transmitted exclusively from fathers who have extremely long expanded repeats with repeat contraction.

The long expansion allele in fathers showed wide variation in repeat length, suggesting repeat instability or somatic mosaicism in blood samples (Figs. 1b, 2a and Additional file 2: Table S2). This is not merely a technological artifact of long-read sequencing technologies because DNA extracted from the LCL established from the F4-father did not show such high variability (Additional file 1: Fig. S8 and Additional file 2: Table S2). Consistently, this repeat instability or somatic mosaicism was confirmed by Southern blot analysis in blood samples from the F2-father, but not in the LCLs from the F3-father or F4-father (Fig. 3b). In the RP-PCR for the F2-father, we detected weak and rapidly diminished signals of sawtooth pattern (Additional file 1: Fig. S1). The F2-father had a wide variation in repeat copy numbers, with a relatively high proportion of reads in the disease-causing range (37.1% in the F2-father compared with 6.5%–12.8% in other fathers) (Fig. 1b and Additional file 2: Table S2). This scanty sawtooth amplification may reflect repeat instability or somatic mosaicism; that is, a relatively high proportion of reads with GGC expansions within the disease-causing range (41‒300 repeats) were amplified by RP-PCR in the F2-father.

To clarify the molecular basis of this repeat instability, we investigated the full GGC repeat expansion sequences in asymptomatic carrier fathers (Fig. 2a). Characterization of HiFi reads did not support any new repeat configuration or expansion-prone sequence within the repeats. Instead, the repeats consisted of long uninterrupted GGC repeats with or without a GGA interruption sequence, as previously reported (Fig. 2a) [3]. The number and position of GGA interruption units was stable across generations (compare patients and fathers of families F2 and F3 in Fig. 2a) and within tissues (F2-father and F3-father in Fig. 2a), despite dynamic changes in GGC repeat units (Fig. 2a). F2-father and F3-father had 2 and 25 GGA repeat interruption units at the 3ʹ end of long stretches of pure GGCs, respectively (Fig. 2a and Additional file 2: Table S3). These GGA interruptions located at the 3ʹ end of the repeat may lessen GGC repeat instability, as suggested for the CGG repeat expansion in fragile X syndrome [22, 23]. In fact, the variation in repeat length was small in the F3-father with 25 GGA interruption units compared with the three other fathers, as indicated by the small standard deviation (SD) and interquartile range (IQR) (106.2 vs. 168.6–205.5 (SD) and 186.8 vs. 298.5–329.0 (IQR)) (Fig. 1b and Additional file 2: Table S2). This is not the case in the F2-father, with 2 GGA repeat interruption units. However, the F2-father had two prominent cell populations with different repeat copy-numbers of approximately 230 and 580 (Figs. 1b and 3b), which may still suggest that two GGAs may stabilize the repeat. Notable also is the ACCGAGAAGATGCCCGCCCTGC insertion event at the 3ʹ end of GGCs in the F1-patient (Fig. 2a). This insertional mutation may also impact repeat instability in the F1-patient during intergenerational transmission. Further studies are needed to evaluate this interruption unit hypothesis.

Recent studies suggest that *NOTCH2NLC* expansion-derived transcripts can produce repeat-containing RNA foci and/or are translated into a toxic polyglycine-containing protein (polyG protein) [12, 13]. We observed consistently that the GGC RNA-positive nuclear inclusions were only formed in LCLs from the affected patients, with possible formation of G4 foci in transcripts from the disease-causing allele. The gain of 5-mC, detected in this study and by others, could possibly abolish the expression of toxic polyG protein/repeat-containing transcripts through epigenetic transcriptional silencing [11, 12]. Deng et al. proposed that *NOTCH2NLC* repeat expansions have a disease-causing range, of 41–300 repeats [12]. Above these repeat numbers, expanded GGCs likely reach a DNA methylation threshold. Currently, we do not know if a clear boundary number separates the hypermethylated allele from the hypomethylated allele. Moreover, we also note that some reads with disease-causing alleles (median repeat length of 140) in the F4-patient were hypermethylated despite having the same repeat length as some hypomethylated reads (Additional file 1: Fig. S9), suggesting some degree of stochastic epigenetic change. These unambiguous epigenetic factors may modify clinical expression.

We discovered the transitional zones of DNA methylation (Fig. 4c and Additional file 1: Fig. S7), which separate the hypomethylated and hypermethylated regions in the normal allele. Interestingly, two transposable elements (LINE L2 family L2c and SINE MIR family MIRb) are located at or nearby the transitional zones (Fig. 4c). Therefore, these transposable elements may form a DNA methylation boundary, as recently reported for the mouse B2 SINE family of elements [24]. These possible DNA methylation boundaries may play a role as a promoter safeguard for *NOTCH2NLC*, which can inhibit the spreading of DNA methylation to the transcriptional regulatory region of *NOTCH2NLC* to ensure expression of the normal allele. Further studies are needed to validate these complex methylation patterns and evaluate these epigenetic scenarios.

Long-read sequencing (LRS) has many advantages for investigating long-range methylation profiles as demonstrated in this study, but some technical limitations remain. Detection of DNA methylation by SMRT sequencing is indirect and relies on polymerase kinetics. Polymerase kinetics must be evaluated cautiously because sequence-specific slowdown of the polymerase can be caused by not only DNA methylation but also stable DNA secondary structures, such as hairpins and G4 structures, and are sequence-context dependent. In fact, the degree of polymerase slowdown is strand-dependent. We observed more prominent slowdown of replication rate in the reverse strand than in the forward strand (compare st = 0 (forward strand) and st = 1 (reverse strand) in Additional file 1: Fig. S4). While Nanopore technology can directly detect 5-mC, the high sequencing error rates of this technology should be taken into consideration [25]. In addition to random sequencing errors, we reported non-random and local DNA sequence context-specific errors at repetitive regions [26]. These errors may result in the miscalling of 5-mC or canonical C. Hence, cross validation using different technologies, including Southern blot, PacBio and Nanopore methylation analysis, is necessary, as demonstrated in this study.

## Conclusions

Investigation of epigenetic status in repetitive elements is challenging because of the incomplete reference sequence and the difficulty in correctly mapping reads (low mappability). This study highlights that long-read sequencing is highly useful in examining the epigenetic landscape of repetitive elements by combining two different long-read sequencing technologies.

## Methods

### Subjects

Four affected individuals with *NOTCH2NLC* repeat expansion and their clinically asymptomatic parents were enrolled in this study. All affected individuals were examined by professional neurologists. Brain MRI and/or skin biopsy were conducted for the clinical diagnosis of NIID. Cognitive and executive functions were evaluated using the Mini Mental State Examination (MMSE), Japanese version of the Montreal Cognitive Assessment (MoCA-J), or Frontal Assessment Battery (FAB). Nerve conduction study was carried out to evaluate the presence of neuropathy. *NOTCH2NLC* repeat expansion was examined by RP-PCR and AL-PCR as described below.

### RP-PCR

RP-PCR was performed according to a previously described protocol [3]. The primer mix contained the following three primers: 5ʹ-FAM-GGCATTTGCGCCTGTGCTTCGGACCGT-3ʹ, 5ʹ-CAGGAAACAGCTATGACCTCCTCCGCCGCCGCCGCC-3ʹ, 5ʹ-CAGGAAACAGCTATGACC-3ʹ.

RP-PCR products were resolved and visualized using a 3500xL Genetic Analyzer (Thermo Fischer Scientific) and analyzed using GeneMapper software (Thermo Fisher Scientific).

### AL-PCR

AL-PCR was performed as previously described [3]. The following PCR primers were used: 5ʹ-VIC-CATTTGCGCCTGTGCTTCGGAC-3ʹ and 5ʹ-AGAGCGGCGCAGGGCGGGCATCTT-3ʹ. AL-PCR products were resolved and visualized using a 3500xL Genetic Analyzer (Thermo Fisher Scientific) and analyzed using GeneMapper software (Thermo Fisher Scientific).

### Nanopore long-read sequencing with Cas9-mediated PCR-free enrichment

CRISPR/Cas9 digestions and library preparations were performed according to the manufacturer’s instructions with the SQK-LSK109 kit (Oxford Nanopore Technologies). Briefly, 5 µg of genomic DNA was treated with Quick calf intestinal phosphatase for 10 min at 37 °C to prevent adapter ligation with the off-target DNA fragment ends, followed by heat inactivation at 80 °C for 3 min. Two target specific-crRNAs on opposite strands (forward and reverse orientations) were designed to flank the *NOTCH2NLC* GGC repeat (chr1:149390803-149390842 from the human reference genome hg38). Approximately 4 kb of genomic DNA fragment was targeted by the double Cas9 digestion (chr1:149389497-149393469). The Mixture of the two Alt-R CRISPR-Cas9 crRNAs (5ʹ-UUCUUAGCCCACUUGUACCCAGG-3ʹ and 5ʹ-GGAGCACUCAAAAGUUUAGAAGG-3ʹ; 10 µM each) and the transactivation crRNA (tracrRNA; 10 µM) in duplex buffer (Integrated DNA Technologies) were denatured at 95 °C for 5 min and cooled to room temperature for 5 min to prepare crRNA:tracrRNA duplexes. The duplexes were incubated at room temperature for 30 min with Alt-R HiFi Cas9 nuclease V3 (Integrated DNA Technologies) to generate ribonucleoprotein (RNP) complexes. Next, dephosphorylated genomic DNA and Cas9 RNP were mixed for target cleavage and simultaneously tailed with dATP for Nanopore adapter ligation using NEB Taq polymerase. The CRISPR/Cas9 digestion and dA-tailing reaction were performed at 37 °C for 60 min and then inactivated at 72 °C for 5 min. Next, Nanopore adapter ligation mix was added to the CRISPR/Cas9-cleaved and dA-tailed sample. Unligated adapters and short DNA fragments were removed with a 0.3 × sample volume of AMPure XP beads (Beckman Coulter), including a washing step with long-fragment buffer (Oxford Nanopore Technologies), before elution in elution buffer (Oxford Nanopore Technologies). Then, sequencing buffer and loading beads were added to the DNA library, which was sequenced with a MinION sequencer, using FLO-MIN106D (R9.4.1) flow cells.

### Repeat analysis using nanopore long-read sequencing data

Target sequencing data from the MinION sequencer was analyzed as previously described [3, 26]. In short, the raw data were base-called and processed into fastq files, using MinKNOW (v.18.12.9). Reads were aligned to the human reference genome hg38 using LAST (http://last.cbrc.jp), and tandem repeat genotyping compared with the hg38 human reference genome (13 copies in the hg38) was carried out using tandem-genotypes v1.3.0 (https://github.com/mcfrith/tandem-genotypes).

### PacBio No-Amp targeted sequencing

No-Amp targeted enrichment and library preparations were performed in accordance with the manufacturer’s instructions (Pacific Biosciences). Briefly, 5 µg of genomic DNA was treated with shrimp alkaline phosphatase (NEB) for 1 h at 37 °C to prevent adapter ligation with the off-target DNA fragment ends, followed by heat inactivation at 65 °C for 10 min. The same crRNAs (5ʹ-UUCUUAGCCCACUUGUACCCAGG-3ʹ and 5ʹ-GGAGCACUCAAAAGUUUAGAAGG-3ʹ) were used as for Nanopore Cas-mediated PCR-free enrichment. The two crRNAs were annealed to tracrRNA separately in duplex buffer (Integrated DNA Technologies), and then pooled in an equimolar mixture. The resultant crRNA:tracrRNA duplexes were incubated at 37 °C for 10 min with Cas9 Nuclease, S. pyogenes (NEB), to generate RNP complexes. Dephosphorylated genomic DNA was mixed with Cas9 RNP for Cas9 digestion, and then purified using a 0.45 × sample volume of AMPure PB beads (Pacific Biosciences). PacBio hairpin barcoded adapter (Pacific Biosciences) was ligated to the Cas9 cleavage sites using T4 DNA Ligase (Thermo Fisher Scientific) at 16 °C for 2 h. The SMRTbell libraries were pooled for multiplexed sequencing, and then purified with a 0.45 × sample volume of AMPure PB beads (Pacific Biosciences). Exonuclease digestion using Exonuclease III (NEB) and enzymes A, B, C and D (Pacific Biosciences) was performed to removed failed ligation products. After the digestion reaction, the SMRTbell library was treated with trypsin for exonuclease removal (Sigma-Aldrich) and purified twice using 0.45 × and 0.42 × sample volumes of AMPure PB beads (Pacific Biosciences). The sequencing primer, v4 (Pacific Biosciences), was conditioned at 80 °C for 2 min, and then annealed to the SMRTbell library at 20 °C for 1 h. After primer annealing, Sequel II DNA Polymerase 2.0 (Pacific Biosciences) was incubated with the SMRTbell template at 30 °C for 4 h to prepare polymerase-bound SMRTbell complex. The SMRTbell DNA/polymerase complex was then purified using a 0.6 × sample volume of AMPure PB beads (Pacific Biosciences). The purified complex was loaded onto the Sequel II SMRT Cell 8 M (Pacific Biosciences). Samples were sequenced on the PacBio Sequel II System using a Sequel II Sequencing Kit 2.0 (Pacific Biosciences), and data were collected for 30 h. One Sequel II SMRT Cell 8 M was used to sequence three samples.

### Repeat analysis using No-Amp data

No-Amp data analysis was carried out using PacBio RepeatAnalysisTools (https://github.com/PacificBiosciences/apps-scripts/tree/master/RepeatAnalysisTools) according to instructions (Pacific Biosciences). Circular consensus sequencing (CCS) was generated using the following command:

- “ccs < inSubreads.bam >  < outCCS.bam > -j 32 --disable-heuristics --draft-mode full”.

To demultiplex No-Amp samples, demultiplex barcodes application was used as follows:

- “lima --same --ccs --split-bam-named --peek-guess -j 8 < outCCS.bam >  < barcodes.fasta >  < outDemuxed.bam > ”.

Alignment to the human reference genome hg38 was carried out using the following command:

- “pbmm2_extention.sh < human_hg38.fasta >  < outDemuxed.bam >  < mapped.ccs.bam > ”.

Repeat analysis of CCS results, such as a waterfall-style plot, was carried out using the scripts provided in RepeatAnalysisTools (https://github.com/PacificBiosciences/apps-scripts/tree/master/RepeatAnalysisTools).

### Polymerase kinetics analysis using No-Amp data

Polymerase kinetics were included in PacBio consensus reads by running ccs v6.0.0: “ccs --hifi-kinetics < subreads.bam >  < hifi.bam > ”. Consensus reads were demultiplexed by sample barcode using lima v2: “lima --ccs --split-named < hifi.bam >  < demux.hifi.bam > ”. For each sample, reads were mapped to GRCh38 (GCA_000001405.15) using pbmm2 v1.4.0 with HiFi parameterization: “pbmm2 align --preset = HiFi < reference.fasta >  < sample.hifi.bam >  < mapped.sample.hifi.bam > ”. Using the mapped GRCh38 region found by alignment as a guide sequence, the on-target reads were clustered into alleles using pbaa v0.1.2: “pbaa cluster < GRCh38_loci.guide.fa >  < sample.hifi.bam >  < output_prefix > ”. Polymerase kinetics were averaged by position across the reads spanning each allele. Average replication cycle time per base was measured as the sum of the average inter-pulse duration (fi tag: forward orientation; ri tag: reverse orientation) and pulse width (fp tag: forward orientation; rp tag: reverse orientation) by strand (forward strand: st = 0; reverse strand: st = 1). Cumulative replication cycle time by allele position was calculated as the sum of the average single-base replication cycle times.

### DNA methylation analysis using nanopore long-read sequencing data

We used guppy (v3.5.2) basecaller to detect 5-methylcytosine (5-mC). Specific basecalling model for modified bases was processed using the configuration file named “dna_r9.4.1_450bps_modbases_dam-dcm-cpg_hac.cfg”. The modified base information was written as a part of fast5 file output. Estimated probabilities of canonical C (unmodified) and 5-mC (modified) were described as integers in the range of 0–255, which represents likelihood in the range of 0%–100%. For example, scores of 255 and 192 for 5-mC indicate likelihoods of 100% (255/255) and 75% (192/255), respectively, of being 5-mC. We set a threshold of > 128 as a modified base (5-mC) in this manuscript. Reads were aligned to the human reference genome hg38 using minimap2 (https://github.com/lh3/minimap2). Then, we used the custom program methylstat for assigning the modified base information from guppy to the genomic position of aligned reads with minimap2. The modified base information from methylstat output was summarized and used for statistical testing for methylation calling at the respective genomic positions (Fisher’s exact test under the null hypothesis of no chance of being methylated) using the custom program methylcall. The methylcall program can also output the percent methylation score [5-mC/(canonical C + 5-mC)] to detect DNA methylation using the “--rate” option. In this study, we set the cut-off value at 20% for methylation calling with the “--rate 0.2” option. Read-level plot showing methylation patterns (in-silico bisulfite-like conversion) were generated using the custom program ont2bisul and visualized using the Integrative Genomics Viewer (IGV). In this custom program, cytosines with 5-mC scores of ≤ 128 were converted to thymine (T) and adenine (A) for forward and reverse reads, respectively, whereas methylcytosine (cytosines with 5-mC scores of > 128) was not converted.

For multi-sample comparison, the publicly available methylkit software for high-throughput bisulfite sequencing experiments was applied to the Nanopore long-read sequencing data [21]. Input file for methylkit was prepared from methylcall output using the custom script mtcall2mtkit.

The methylstat, methylcall, ont2bisul and mtcall2mtkit custom programs are available at https://github.com/bitsyamagu/methyl-stat/tree/main.

### DNA methylation analysis within the GGC repeat sequence

PacBio amplicon analysis (pbaa) tool (https://github.com/PacificBiosciences/pbAA) was used to cluster and generate high-quality consensus sequences from HiFi reads (pbaa consensus) as described above. Nanopore reads from Cas9-mediated PCR-free enrichment were classified into non-expanded allele (tandem-genotypes call < 50) and expanded allele (tandem-genotypes call ≥ 50) based on GGC repeat copy number from tandem-genotypes call, and then aligned to the PacBio pbaa consensus sequence using minimap2 (https://github.com/lh3/minimap2). *NOTCH2NLC* is located within a segmental duplication highly homologous to four other genomic regions. To improve the mapping accuracy, the four homologous sequences (chr1:119989248-120093730, chr1:120705588-12081063, chr1:146139729-146254754 and chr1:148590677-148705497) were added as decoy sequences before minimap2 alignment. Correct alignments of Nanopore reads to *NOTCH2NLC* region were supported by the *NOTCH2NLC* specific insertion of an *AluYa5* (chr1:149392790-149393094) [4].

### Southern blot analysis

For evaluating *NOTCH2NLC* repeat expansion, 5 µg of genomic DNA was digested with NheI (NEB). For DNA methylation analysis, 15 µg of genomic DNA was initially digested with NheI (NEB) and purified by phenol–chloroform extraction. Purified DNA was divided into three parts and subjected to secondary digestion with either methylation sensitive or insensitive isoschizomers HpaII and MspI (NEB), respectively, with no secondary digestion. Digested DNA was separated on 0.8% agarose gels (w/v) in 1.0 × Tris/borate/EDTA buffer at 4 °C for 2 h, and then transferred to positively-charged nylon membranes by capillary transfer. DNA fragments were fixed to the membranes using the autocrosslink mode of the Stratalinker UV Crosslinker 2400 (Stratagene). The digoxigenin (DIG)-labeled probe was generated by PCR amplification from the DNA fragment cloned into TOPO qCR 2.1 vector in accordance with the manufacturer’s instructions (Roche). The following PCR primers were used for generating the hybridization probe: 5ʹ-AACGGATGACACTCCAAAGG-3ʹ and 5ʹ-TCCTGCTTCATAGGTGAAGAGAC-3ʹ. Prehybridization was performed at 37 °C for 1 h in DIG Easy Hyb buffer. Hybridization was performed at 37 °C overnight in DIG Easy Hyb buffer containing the DIG-labeled unique PCR probe. After hybridization, membranes were washed twice at room temperature in 2 × SSC/0.1% SDS for 5 min, followed by two 15-min washes in 0.5 × SSC/0.1% SDS at 68 °C. The DIG-labeled probe was visualized by chemiluminescence detection using anti-DIG antibodies conjugated with alkaline phosphatase (anti-DIG-AP) and its chemiluminescence substrate CSPD-star (Roche). Briefly, membranes were blocked for 30 min in 1 × blocking solution, and then incubated for 30 min in antibody solution (75 mU/mL of anti-DIG-AP), followed by two 15-min washes in washing buffer (0.1 M maleic acid, 0.15 M NaCl, 0.3% Tween 20) at room temperature. Finally, the chemiluminescence reaction was performed using CSPD-Star and visualized using a ChemiDoc Touch imaging system (Bio-Rad).

### RNA fluorescence in situ hybridization (FISH) and immunofluorescence

RNA FISH and immunocytochemistry were performed as previously described [20]. In brief, the lymphoblastoid cell lines (LCLs) derived from F3 and F4 families were fixed with 4% paraformaldehyde in phosphate-buffered saline (PBS), and then washed with diethylpyrocarbonate-treated PBS (DEPC-PBS; 3 × 20 min). For permeabilization, cells were incubated with 70% ethanol at 4 °C overnight. After several washes, LCLs were plated on coverslips coated with 0.1% poly-l-lysine. After cell attachment and several washes with 1 × DEPC-PBS, LCLs were blocked in hybridization solution (40% formamide, 10% dextran sulfate, 2 mM vanadyl sulfate and 0.5 mg/mL yeast tRNA in 2 × SSC (300 mM NaCl, 30 mM sodium citrate)) for 1 h at 37 °C, and then incubated with 1 nM Cy5-(CCG)_8_ probe in hybridization solution at 37 °C overnight. Thereafter, LCLs were washed sequentially with 40% formamide in 2 × SSC, 2 × SSC and 0.2 × SSC at 37 °C (2 × 20 min). Samples were washed with 1 × PBS, incubated with 1% bovine serum albumin and 0.1% Triton-X in PBS (blocking solution) for 1 h at room temperature, and then treated with anti-p62 (1:1,000; GP62-C, PROGEN Biotechnik) and anti-ubiquitin (1:1,000; ZO458, Dako) antibodies, as markers of inclusion bodies, diluted in blocking solution overnight at 4 °C. For staining G-quadruplex (G4) structures, LCLs were incubated with an antibody against the 6X His epitope tag (1:1,000; ab18184, Abcam) present on BG4 (1:800, 1.0 μg/mL) [20]. After several rinses, diluted secondary antibodies Alexa 488-conjugated goat anti-guinea pig (1:1,000; A-11073, Invitrogen) and Alexa 594-conjugated donkey anti-rabbit (1:1,000; A-21207, Invitrogen) or Alexa 594-conjugated donkey anti-mouse (1:1,000; A-21203, Invitrogen) were reacted with LCLs overnight at 4 °C. After several washes and 4ʹ,6-diamidino-2-phenylindole (DAPI) staining, LCLs were mounted with Vectashield (Vector Laboratories). Immunofluorescent images of each sample were obtained using a confocal laser-scanning microscope (Leica TCS SP8). The number of p62/ubiquitin double-positive intranuclear inclusions in LCLs was counted and calculated as a ratio.

### Reverse-transcription quantitative PCR (RT-qPCR)

Total RNA was purified from LCLs using the RNeasy Mini Kit (Qiagen) in accordance with the manufacturer’s protocol. The RNA was reverse-transcribed into single-stranded cDNA using oligo(dT) primers (Promega) and Moloney murine leukemia virus reverse transcriptase (Invitrogen), and then subjected to RT-qPCR with gene-specific primers, performed as previously described, using iQ SYBR Green Supermix (Bio-Rad) [20]. Gene expression was analyzed using the comparative threshold cycle (ΔCT) method and normalized to *GAPDH* expression, and then further relative to a healthy mother sample. The following primers were used for RT-qPCR:

Notch2nlc (Fw): 5ʹ-GATCTTTCCAAAGAGAATTCTGTATCTC-3ʹ; Notch2nlc (Rv): 5ʹ-GAGAGCCACATGGCTGACTT-3ʹ; GAPDH (Fw): 5ʹ-CTGGGCTACACTGAGCACC-3ʹ; GAPDH (Rv): 5ʹ-AAGTGGTCGTTGAGGGCAATG-3ʹ.

### Statistical analyses

Statistical analysis was performed using R, version 3.6.2. For Fig. 6c, R function of cor.test with parameter method = "pearson" was used (https://www.r-project.org/). For Table S6, Methylkit function of calculateDiffMeth was used to extract differentially methylated bases by Fisher’s exact test and calculate *p*-values. The sliding linear model (SLIM) method was used to calculate *q*-values, corrected for multiple hypothesis testing, and values of *q* < 0.01 were considered significant. One-way analysis of variance (ANOVA) with post-hoc Bonferroni’s multiple comparison test was used for analysis of RT-qPCR data (Fig. 5c). Data were expressed as the mean ± standard error of the mean (SEM), and *p* < 0.05 represented a statistically significant difference.

## Supplementary Information

**Additional file 1: Fig. S1**. De novo *NOTCH2NLC* repeat expansion are suspected by RP-PCR; **Fig. S2**. Long repeat expansion of asymptomatic carrier fathers was confirmed by Southern blot analysis; **Fig. S3**. Maternal transmission of non-expanded allele as indicated by AL-PCR; **Fig. S4**. Polymerase kinetics study suggesting DNA base modification in asymptomatic carrier fathers; **Fig. S5**. Nanopore methylation analysis revealed gain of 5-mC in the asymptomatic carrier father (F2-father); **Fig. S6**. Consensus sequence of expanded and non-expanded alleles using PacBio HiFi reads; **Fig. S7**. Read-based methylation plots of 12 individuals; **Fig. S8**. Bimodal distribution of non-expanded and expanded alleles in the LCLs from the F4-father, not suggesting somatic mosaicism; **Fig. S9**. Read-based methylation plots for the F4-patient, showing the gain of 5-mC in the small percentage of disease-causing allele (median 162 repeat units); **Fig. S10**. Full unedited image for Fig. 3b and S2.

**Additional file 2: Table S1**. Clinical features of four individuals with disease-causing *NOTCH2NLC* repeat expansions; **Table S2**. Summary statistics of Nanopore Cas9-based enrichment sequencing; **Table S3**. PacBio HiFi-read-based repeat composition; **Table S4**. Ratio of LCLs with nuclear inclusions in F3 and F4 families; **Table S5**. Percent methylation score in the F2 family; **Table S6**. Differentially methylated regions identified by Methylkit.

## Data Availability

The methylstat, methylcall, ont2bisul and mtcall2mtkit custom programs are available at https://github.com/bitsyamagu/methyl-stat/tree/main.

## Abbreviations

NIID: Neuronal intranuclear inclusion disease
LRS: Long-read sequencing
5-mC: 5-Methylcytosine
No-Amp: PacBio no-amplification targeted sequencing method
SINE: Short interspersed nuclear elements
LINE: Long interspersed nuclear elements
NGS: Next-generation sequencing
LCLs: Lymphoblastoid cell lines

## References

[1] Sone J, Mori K, Inagaki T, Katsumata R, Takagi S, Yokoi S (2016). Clinicopathological features of adult-onset neuronal intranuclear inclusion disease. Brain.

[2] Tian Y, Wang JL, Huang W, Zeng S, Jiao B, Liu Z (2019). Expansion of human-specific ggc repeat in neuronal intranuclear inclusion disease-related disorders. Am J Hum Genet.

[3] Sone J, Mitsuhashi S, Fujita A, Mizuguchi T, Hamanaka K, Mori K (2019). Long-read sequencing identifies GGC repeat expansions in *NOTCH2NLC* associated with neuronal intranuclear inclusion disease. Nat Genet.

[4] Ishiura H, Shibata S, Yoshimura J, Suzuki Y, Qu W, Doi K (2019). Noncoding CGG repeat expansions in neuronal intranuclear inclusion disease, oculopharyngodistal myopathy and an overlapping disease. Nat Genet.

[5] Shi CH, Fan Y, Yang J, Yuan YP, Shen S, Liu F (2021). *NOTCH2NLC* intermediate-length repeat expansions are associated with Parkinson disease. Ann Neurol.

[6] Yuan Y, Liu Z, Hou X, Li W, Ni J, Huang L (2020). Identification of GGC repeat expansion in the *NOTCH2NLC* gene in amyotrophic lateral sclerosis. Neurology.

[7] Sun QY, Xu Q, Tian Y, Hu ZM, Qin LX, Yang JX (2020). Expansion of GGC repeat in the human-specific *NOTCH2NLC* gene is associated with essential tremor. Brain.

[8] Ogasawara M, Iida A, Kumutpongpanich T, Ozaki A, Oya Y, Konishi H (2020). CGG expansion in *NOTCH2NLC* is associated with oculopharyngodistal myopathy with neurological manifestations. Acta Neuropathol Commun.

[9] Ishihara T, Okamoto T, Saida K, Saitoh Y, Oda S, Sano T (2020). Neuronal intranuclear inclusion disease presenting with an MELAS-like episode in chronic polyneuropathy. Neurol Genet.

[10] Okubo M, Doi H, Fukai R, Fujita A, Mitsuhashi S, Hashiguchi S (2019). GGC repeat expansion of *NOTCH2NLC* in adult patients with leukoencephalopathy. Ann Neurol.

[11] Yu J, Deng J, Guo X, Shan J, Luan X, Cao L, et al. The GGC repeat expansion in *NOTCH2NLC* is associated with oculopharyngodistal myopathy type 3. Brain 144(6):1819–1832. 10.1093/brain/awab077 PMC8320266 33693509

[12] Deng J, Zhou B, Yu J, Han X, Fu J, Li X (2021). Genetic origin of sporadic cases and RNA toxicity in neuronal intranuclear inclusion disease. J Med Genet.

[13] Boivin M, Deng J, Pfister V, Grandgirard E, Oulad-Abdelghani M, Morlet B (2021). Translation of GGC repeat expansions into a toxic polyglycine protein in NIID defines a novel class of human genetic disorders: the polyG diseases. Neuron.

[14] McGinty RJ, Mirkin SM (2018). Cis- and trans-modifiers of repeat expansions: blending model systems with human genetics. Trends Genet.

[15] Mitsuhashi S, Frith MC, Mizuguchi T, Miyatake S, Toyota T, Adachi H (2019). Tandem-genotypes: robust detection of tandem repeat expansions from long DNA reads. Genome Biol.

[16] Wenger AM, Peluso P, Rowell WJ, Chang PC, Hall RJ, Concepcion GT (2019). Accurate circular consensus long-read sequencing improves variant detection and assembly of a human genome. Nat Biotechnol.

[17] Wick RR, Judd LM, Holt KE (2019). Performance of neural network basecalling tools for Oxford Nanopore sequencing. Genome Biol.

[18] Naumann A, Hochstein N, Weber S, Fanning E, Doerfler W (2009). A distinct DNA-methylation boundary in the 5'- upstream sequence of the *FMR1* promoter binds nuclear proteins and is lost in fragile X syndrome. Am J Hum Genet.

[19] Xu K, Li Y, Allen EG, Jin P (2021). Therapeutic development for CGG repeat expansion-associated neurodegeneration. Front Cell Neurosci.

[20] Asamitsu S, Yabuki Y, Ikenoshita S, Kawakubo K, Kawasaki M, Usuki S (2021). CGG repeat RNA G-quadruplexes interact with FMRpolyG to cause neuronal dysfunction in fragile X-related tremor/ataxia syndrome. Sci Adv.

[21] Akalin A, Kormaksson M, Li S, Garrett-Bakelman FE, Figueroa ME, Melnick A (2012). methylKit: a comprehensive R package for the analysis of genome-wide DNA methylation profiles. Genome Biol.

[22] Nolin SL, Glicksman A, Ersalesi N, Dobkin C, Brown WT, Cao R (2015). Fragile X full mutation expansions are inhibited by one or more AGG interruptions in premutation carriers. Genet Med.

[23] Eichler EE, Holden JJ, Popovich BW, Reiss AL, Snow K, Thibodeau SN (1994). Length of uninterrupted CGG repeats determines instability in the FMR1 gene. Nat Genet.

[24] Ichiyanagi T, Katoh H, Mori Y, Hirafuku K, Boyboy BA, Kawase M (2021). B2 SINE copies serve as a transposable boundary of DNA methylation and histone modifications in the mouse. Mol Biol Evol.

[25] Bowden R, Davies RW, Heger A, Pagnamenta AT, de Cesare M, Oikkonen LE (2019). Sequencing of human genomes with nanopore technology. Nat Commun.

[26] Mizuguchi T, Toyota T, Miyatake S, Mitsuhashi S, Doi H, Kudo Y (2021). Complete sequencing of expanded *SAMD12* repeats by long-read sequencing and Cas9-mediated enrichment. Brain.

