# Supplementary material for: Father-to-offspring transmission of extremely long NOTCH2NLC repeat expansions with contractions: genetic and epigenetic profiling with long-read sequencing
Source: Clin Epigenetics. 2021 Nov 13;13:204. doi: 10.1186/s13148-021-01192-5 (PMC8590777; doi:10.1186/s13148-021-01192-5)
Supplement: Supplementary file 1 — Additional file 1: Fig. S1. De novo NOTCH2NLC repeat expansion are suspected by RP-PCR; Fig. S2. Long repeat expansion of asymptomatic carrier fathers was confirmed by Southern blot analysis; Fig. S3. Maternal transmission of non-expanded allele as indicated by AL-PCR; Fig. S4. Polymerase kinetics study suggesting DNA base modification in asymptomatic carrier fathers; Fig. S5. Nanopore methylation analysis revealed gain of 5-mC in the asymptomatic carrier father (F2-father); Fig. S6. Consensus sequence of expanded and non-expanded alleles using PacBio HiFi reads; Fig. S7. Read-based methylation plots of 12 individuals; Fig. S8. Bimodal distribution of non-expanded and expanded alleles in the LCLs from the F4-father, not suggesting somatic mosaicism; Fig. S9. Read-based methylation plots for the F4-patient, showing the gain of 5-mC in the small percentage of disease-causing allele (median 162 repeat units); Fig. S10. Full unedited image for Fig. 3b and S2. [file 13148_2021_1192_MOESM1_ESM.pdf]

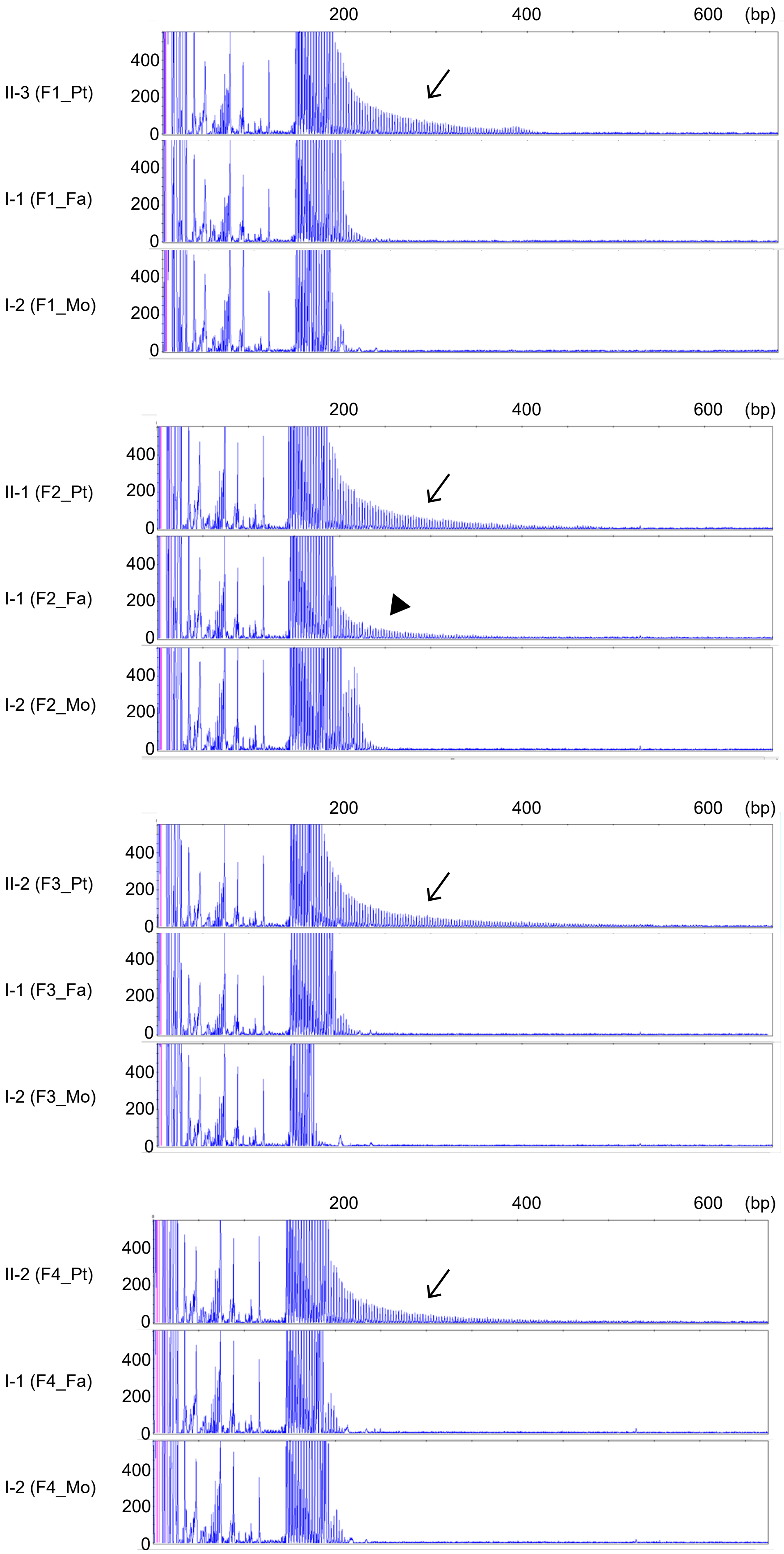

Fig. S1 De novo *NOTCH2NLC* repeat expansion are suspected by RP-PCR. RP-PCR targeting the *NOTCH2NLC* GGC repeat was performed in four sporadic cases and their parents (12 individuals) using genomic DNA extracted from blood samples. The sawtooth pattern was readily detected in the affected individuals (arrow), but not in their parents, except the F2-father, with rapidly waning intensity (arrowhead). Pt: patient; Fa: father; Mo: mother.

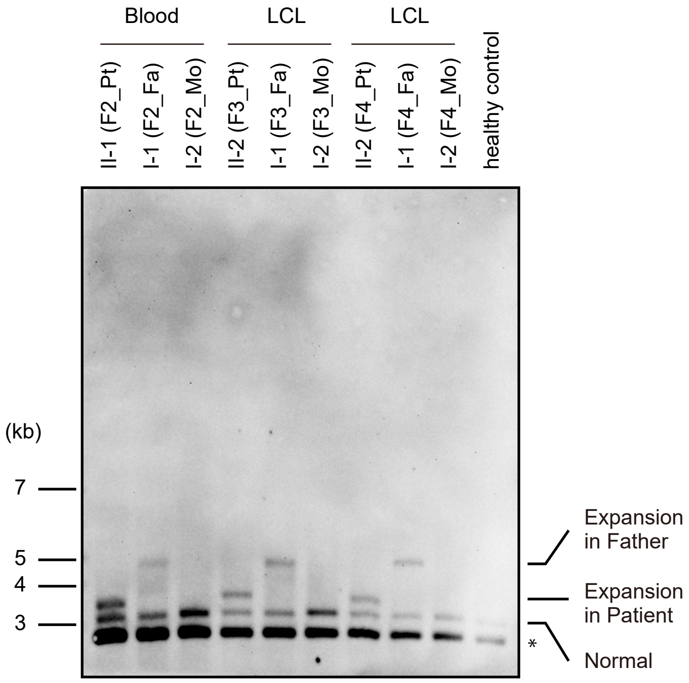

Fig. S2 Long repeat expansion of asymptomatic carrier fathers was confirmed by Southern blot analysis. The genomic DNA extracted from blood samples (F2 family) and LCLs (F3 and F4 families) were digested by NheI. The NheI DNA fragment containing the *NOTCH2NLC* GGC repeat was detected using the same probe described in Fig. 3a. Asterisk: cross hybridization to the *NOTCH2NLC* homologous genes, *NOTCH2*, *NOTCH2NLA*, *NOTCH2NLB* and *NOTCH2NLR*. Pt: patient; Fa: father; Mo: mother.

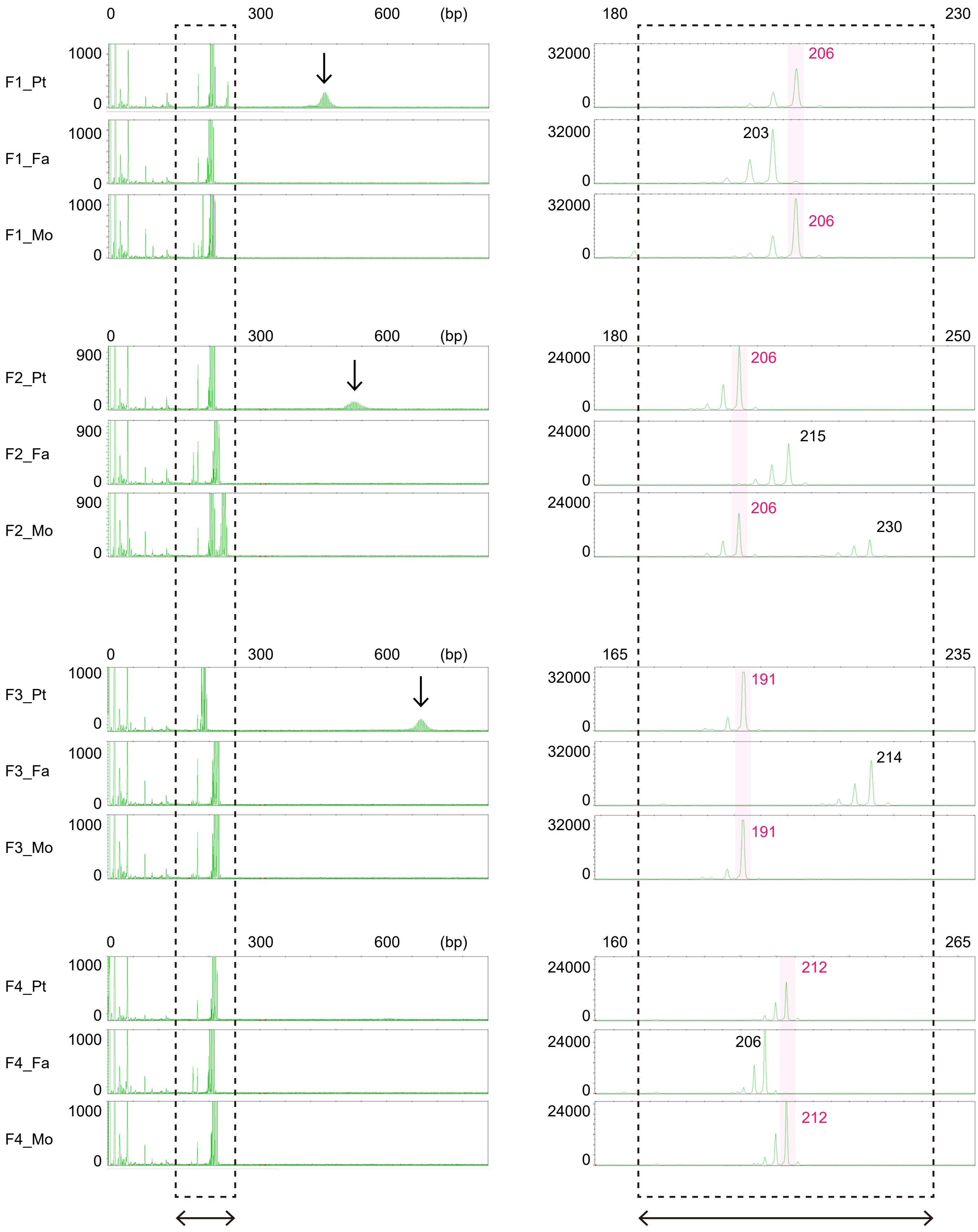

Fig. S3 Maternal transmission of non-expanded allele as indicated by AL-PCR. AL-PCR targeting *NOTCH2NLC* repeat was performed in four sporadic cases and their parents (12 individuals) using genomic DNA extracted from blood samples. AL-PCR can detect expanded allele in F1, F2 and F3-patient (arrow), but not in F4-patient. The zoomed-in images of non-expanded allele (dashed box) were shown on the right panel. Double-headed arrows show the same interval of target DNA fragment (non-expanded allele). The sizes of maternally transmitted non-expanded alleles were shown in red numbers (allele size). Pt: patient, Fa: father, Mo: mother.

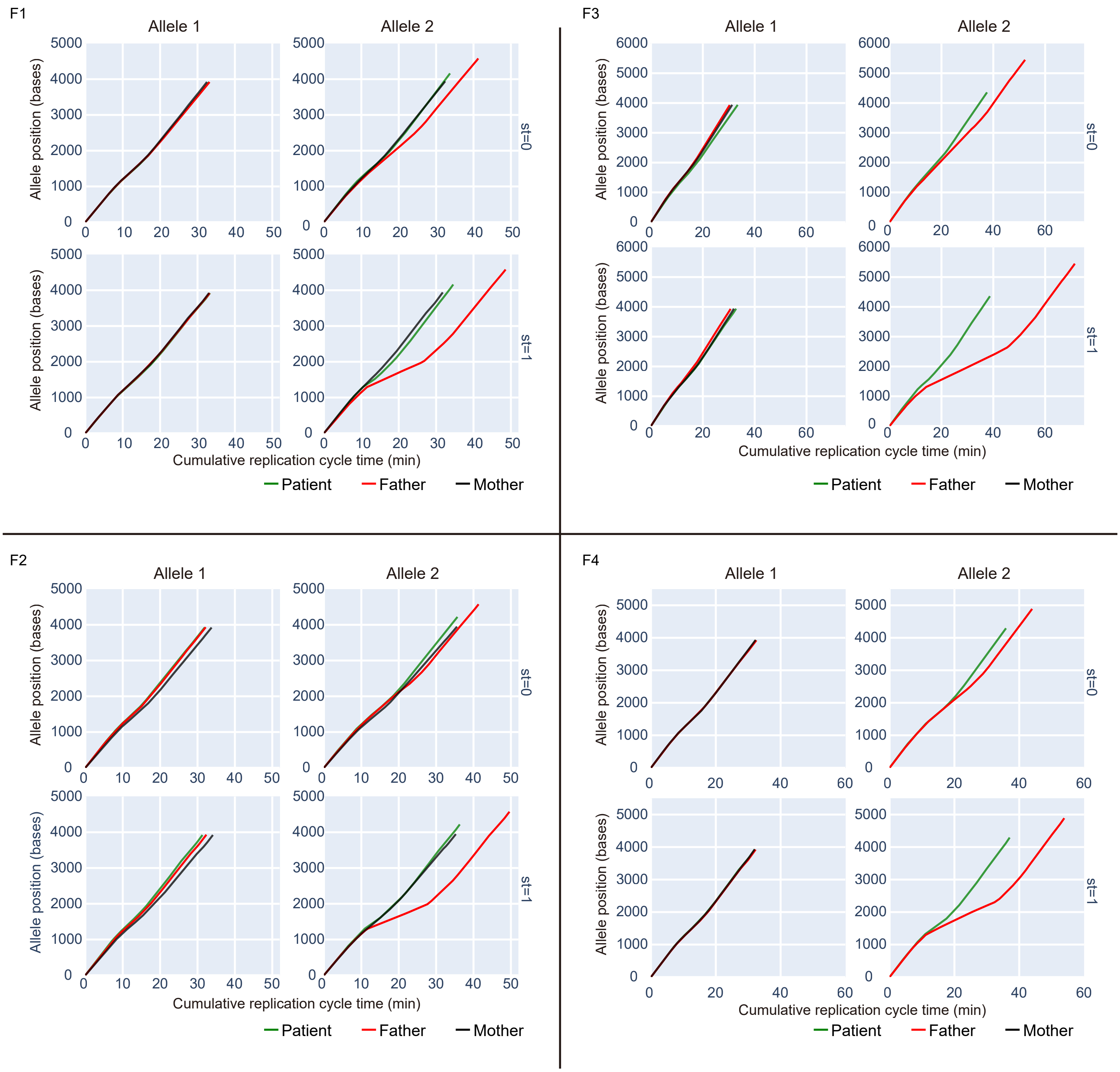

Fig. S4 Polymerase kinetics study suggesting the DNA base modification in asymptomatic carrier fathers. Cumulative replication cycle time of non-expanded and expanded alleles for each template position during the Single Molecule, Real-Time (SMRT) sequencing was shown. st=0, forward strand; st=1, reverse strand. Allele 1: Non-expanded allele, Allele 2: Expanded allele of patients and their fathers, and second non-expanded allele of F1- and F2-mothers. Unphased non-expanded alleles of F3- and F4-mothers were displayed only in allele 1 because two non-expanded alleles with similar repeat sizes could not be separated.

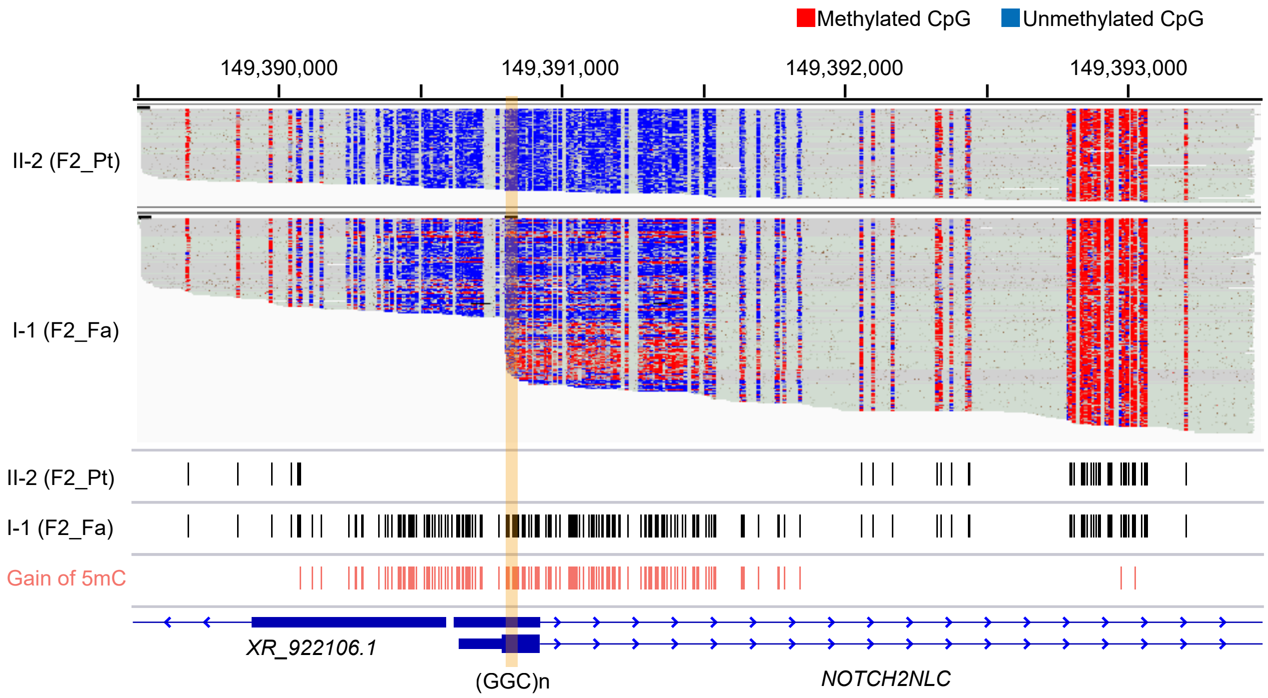

Fig. S5 Nanopore methylation analysis revealed gain of 5-mC in the asymptomatic carrier father (F2-father). Long-range methylation information of individual Nanopore reads was visualized by IGV bisulfite mode (top). The methylation calling by our custom program, methylcall, is shown in the lower part (black vertical bar). Non-overlapping calls between F2-patient and F2-father (Gain of 5-mC) were extracted using bedtools intersect command (https://bedtools.readthedocs.io/en/latest/) and indicated by red vertical bars. Pt: patient; Fa: father.

a

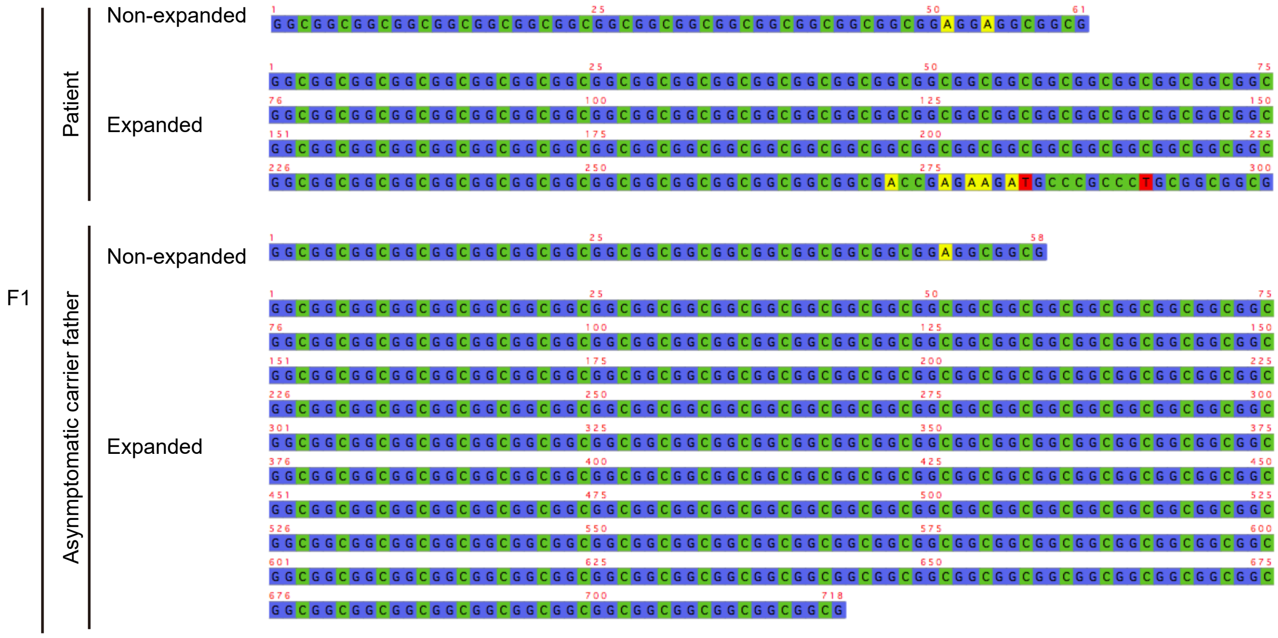

b

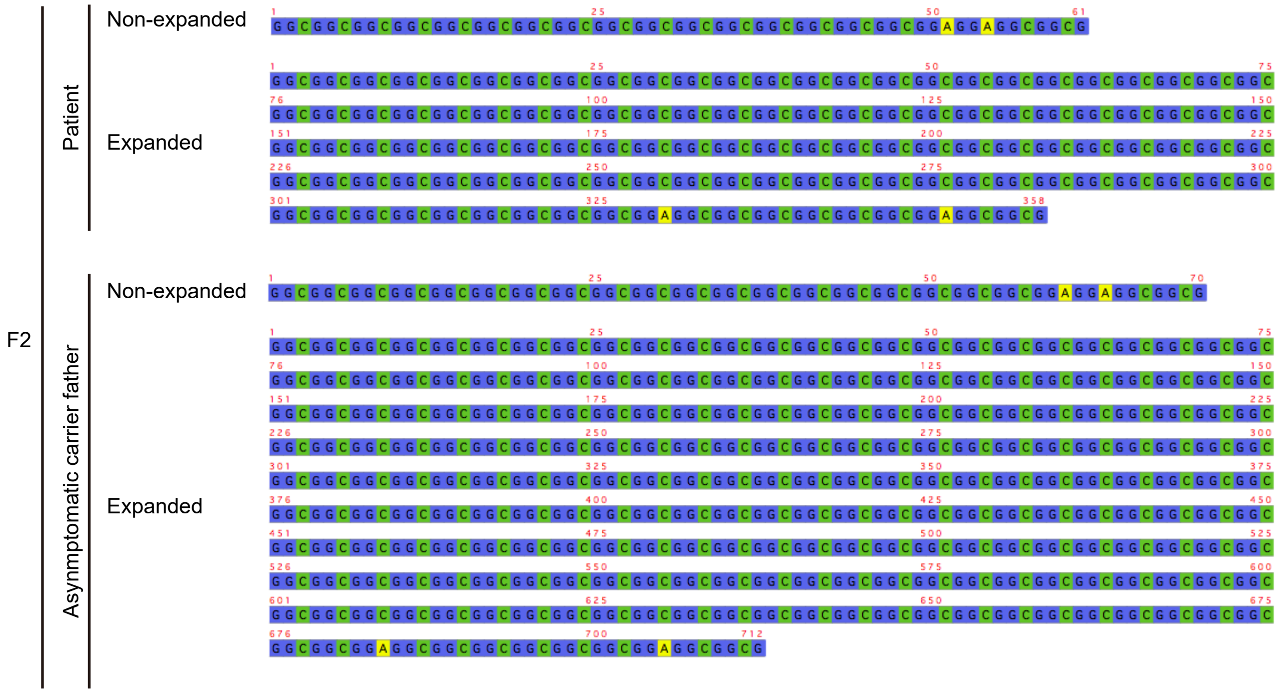

Fig. S6 Consensus sequence of expanded and non-expanded alleles using PacBio HiFi reads. High-quality consensus sequences from HiFi reads were generated using the PacBio Amplicon Analysis (pbaa, https://github.com/PacificBiosciences/pbAA) tool for family F1 (a), F2 (b), F3 (c) and F4 (d).

continued

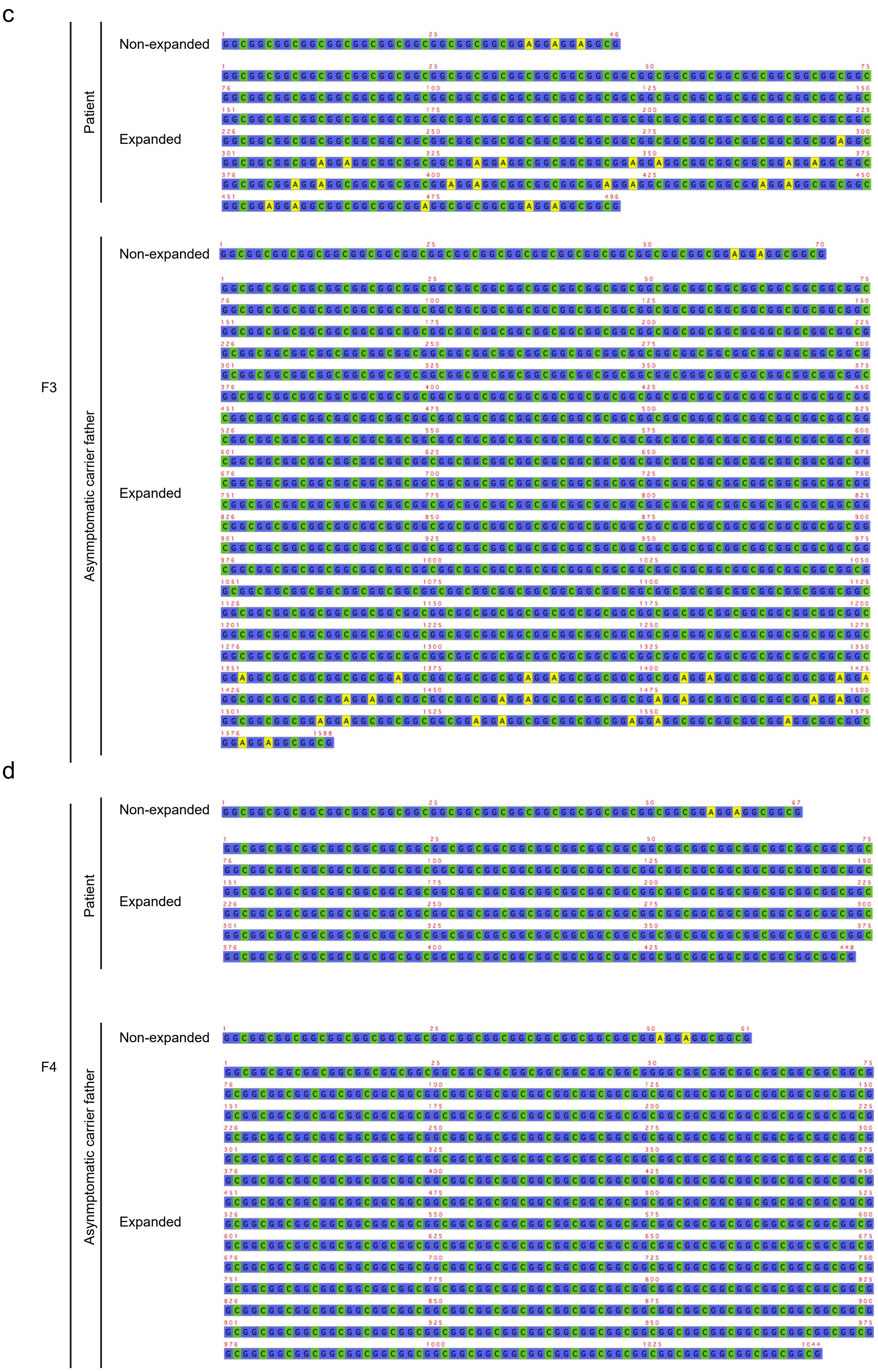
c
Patient
Non-expanded
Expanded
F3
Asymptomatic carrier father
Non-expanded
Expanded
d
Patient
Non-expanded
Expanded
F4
Asymptomatic carrier father
Non-expanded
Expanded

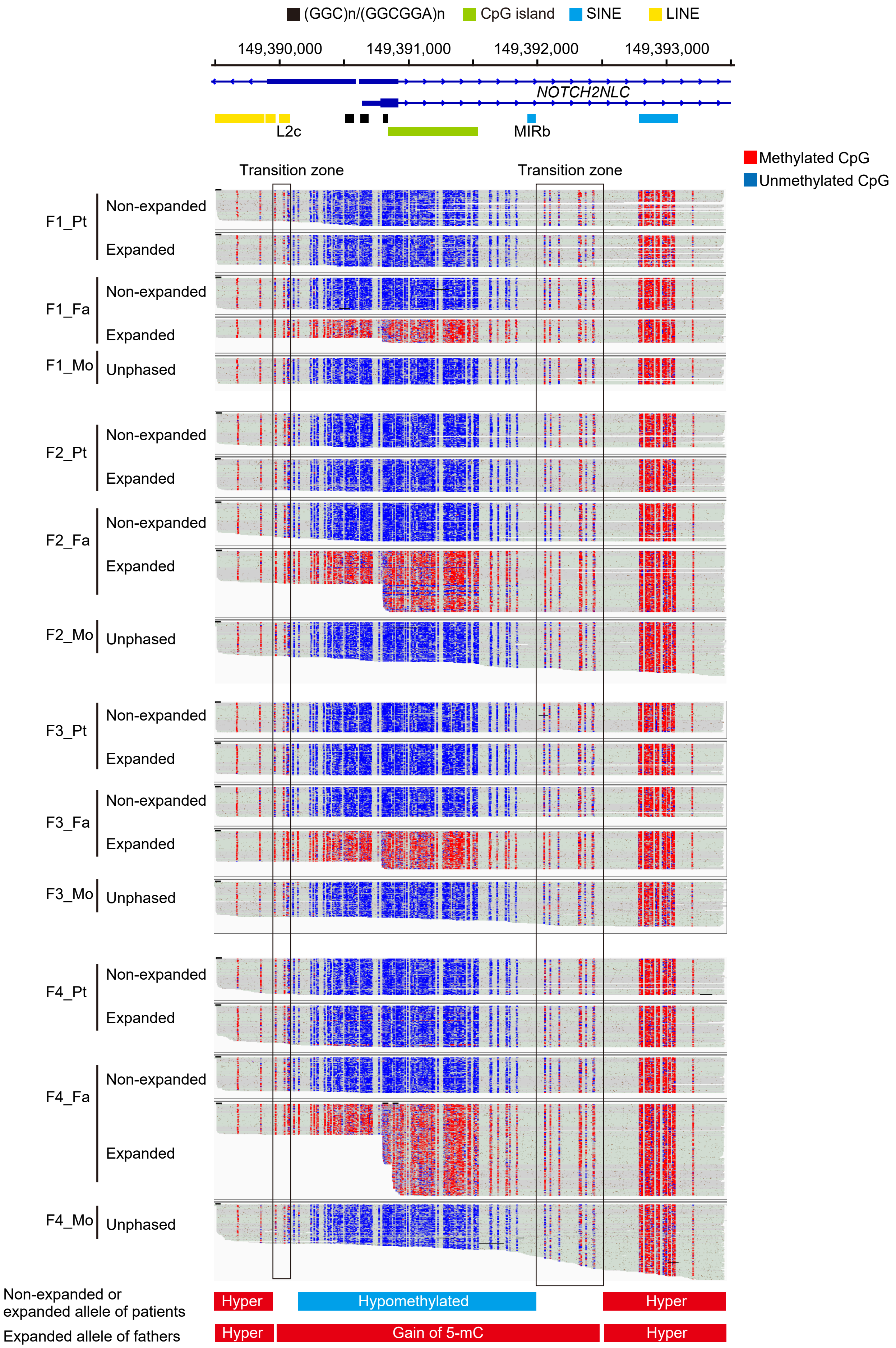

Fig. S7 Read-based methylation plots of 12 individuals. All four families showed similar DNA methylation profiles. Asymptomatic carrier fathers had a different methylation pattern compared with patients and their mothers. The position of transitional zones was conserved in all 12 individuals tested in this study (black-lined rectangles). Pt: patient; Fa: father; Mo: mother. Black, green, blue and yellow rectangles represent (GGC)n/(GGCGGA)n, CpG island, SINE and LINE, respectively.

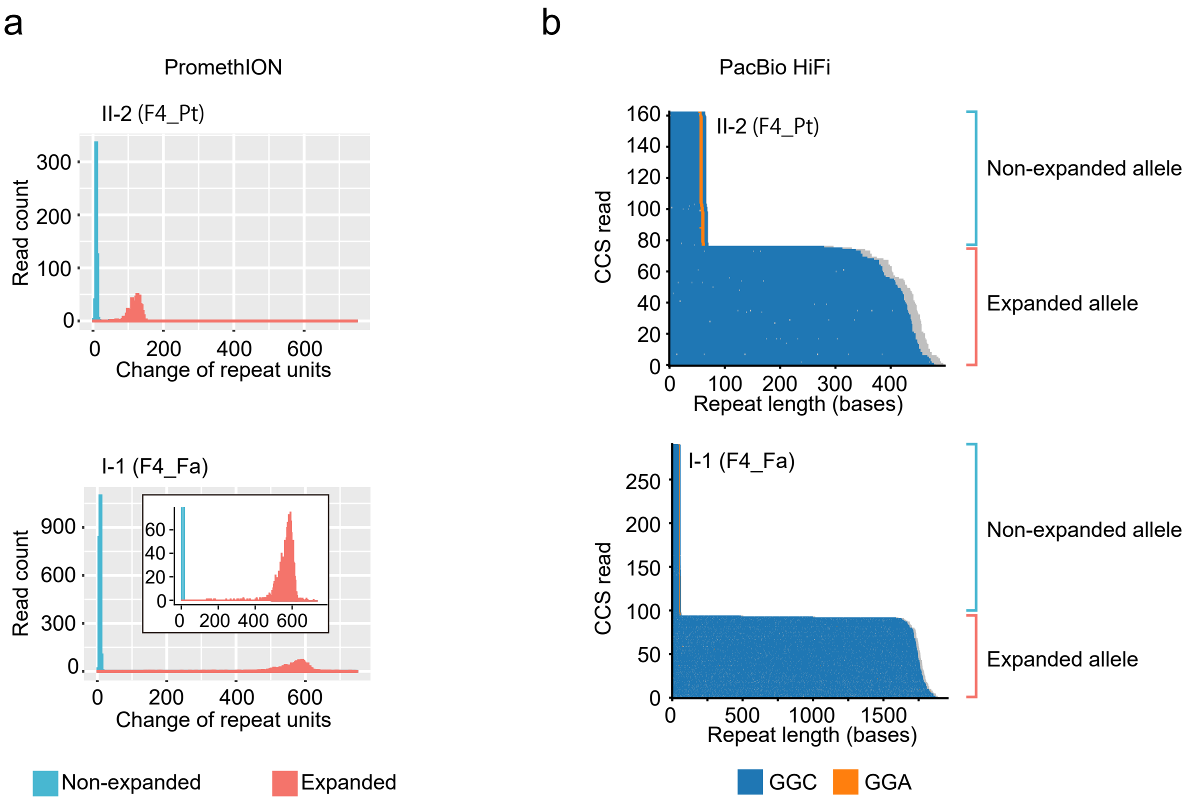

Fig. S8 Bimodal distribution of non-expanded and expanded alleles in the LCLs from the F4-father, not suggesting somatic mosaicism. a Repeat-size evaluation of *NOTCH2NLC* repeat using DNA extracted from LCLs. The copy-number change in the *NOTCH2NLC* repeat relative to the human reference genome (hg38) was estimated from Nanopore reads. Pale blue: non-expanded allele; pale pink: expanded allele. **b** Waterfall plots showing repeat structure in patients and their asymptomatic carrier fathers. The Y-axis shows the number of circular consensus sequence (CCS) reads, whereas the X-axis shows the length of the repeat expansions (bases). GGC and GGA sequences are shown in blue and orange, respectively. Pt: patient; Fa: father.

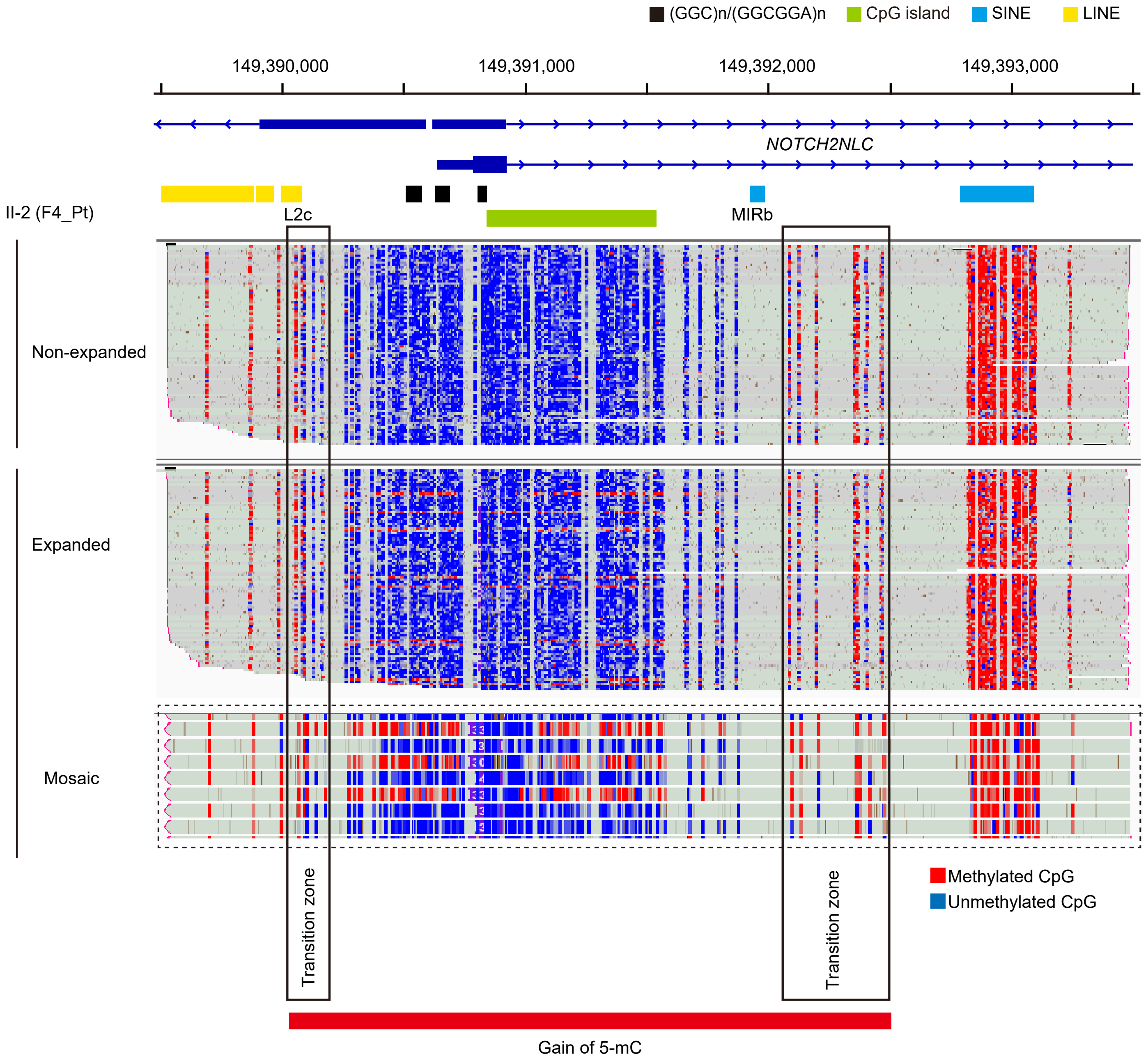

Fig. S9 Read-based methylation plots of F4-patient, showing the gain of 5-mC in small percentage of disease-causing allele (median 162 repeat units). A very small percentage, but certainly some leads are showing hypermethylation. Representative reads with hypo- and hyper-methylated CpG, indicating epigenetic mosaicism, are shown in lower inset (dashed-line boxes). Pt: patient. Black, green, blue and yellow rectangles represent (GGC)n/(GGCGGA)n, CpG island, SINE and LINE, respectively.

F2 (Blood)

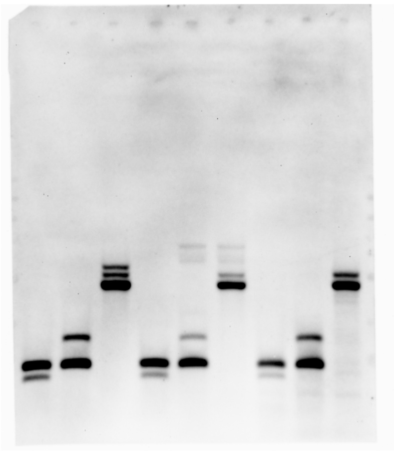

Fig. 3b

F3 (LCL)

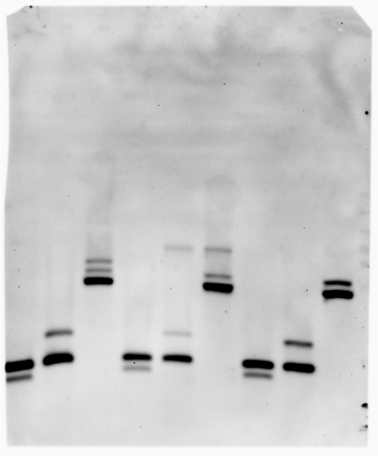

Fig. 3b

F4 (LCL)

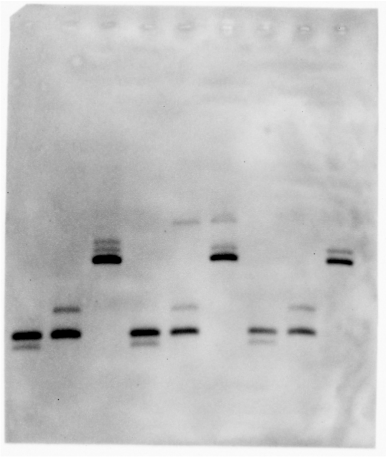

Fig. 3b

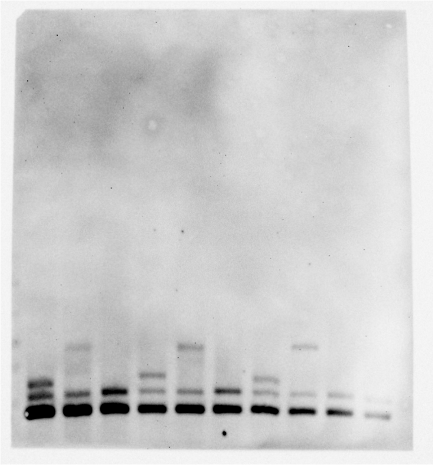

Fig. S2

Fig. S10 Full unedited image for Fig. 3b and S2.
